# Robust hierarchical co-clustering for exploring toxicogenomic biomarkers and their chemical regulators

**DOI:** 10.1038/s41598-025-99568-7

**Published:** 2025-05-14

**Authors:** Mohammad Nazmol Hasan, Md Bahadur Badsha, Md. Nurul Haque Mollah

**Affiliations:** 1 Department of Statistics, Gazipur Agricultural University, Gazipur, 1706 Bangladesh; 2 Center for Applied Bioinformatics, St Jude Children’s Research Hospital, Memphis, TN USA; 3 Bioinformatics Lab., Department of Statistics, University of Rajshahi, Rajshahi, 6205 Bangladesh; 4 Present Address: Sera Prognostics, Inc., Salt Lake City, UT USA

**Keywords:** Computational biology and bioinformatics, Biomarkers

## Abstract

Toxicity measurement of doses of chemicals (DCs) is one of the most important tasks in toxicology studies and the drug discovery and development process. In this issue, toxicogenomic biomarkers are now playing a vital role in measuring the toxicity of DCs. Differentially expressed genes (DEGs) between DCs-treatment and control groups are considered toxicogenomic biomarkers, and associated chemicals are the regulators of DEGs. The co-clustering technique is now used extensively in toxicogenomic research to investigate co-clusters between genomic biomarkers and their chemical regulators. In the literature, there are few approaches to exploring co-clusters. The hierarchical co-clustering (HCoClust) approach is faster, simpler, and more flexible. Nevertheless, it is not robust against outlier data and there is no instruction about separating upregulatory or downregulatory co-clusters, a crucial goal of toxicogenomic data analysis. Therefore, in this article, we proposed a robust HCoClust (rHCoClust) approach and developed an r-package called “rhcoclust” for its implementation. Simulation results showed that the conventional HCoClust and the proposed rHCoClust performed equally well in detecting co-clusters in the absence of outliers, while rHCoClust performed much better than HCoClust in the presence of outliers. However, rHCoClust outperformed the bi-clustering approaches in detecting co-clusters, since bi-clustering methods only work when row and column clusters are equal, and they have no criterion for detecting upregulatory and downregulatory co-clusters. Then rHCoClust was compared with HCoClust through real data analysis and found that rHCoClust performed better than HCoClust. In the case of real data analysis, the proposed method rHCoClust identified top-ranked two DEGs-clusters (*GSTA5, MGST2, GCLC, GCLM, G6PD*) and (*EHHADH, CYP4A1, ANGPT14, CPT1A*) that were significantly expressed by the influence of top-ranked two DCs-clusters (acetaminophen_High _24.hr, nitrofurazone_High_24.hr, methapyrilene_High_24.hr) and (WY.14643_High_24.hr, clofibrate_High_24.hr, gemfibrozil_High_24.hr, benzbromarone_High_24.hr, aspirin_High_24.hr) through the glutathione metabolism (GMP) and PPAR signaling pathway (PPAR-SP) respectively. The literature review also supported these results. Thus, the proposed method would be useful to explore toxicogenomic biomarkers and their chemical regulators from the robustness point of view.

## Introduction

Toxicogenomic studies measure the toxicity of chemical agents at the genome level in an organism. This is one of the most important tasks in toxicology studies as well as in the drug discovery and development process^1–5^. The toxicogenomic studies also examine how the genetic factors of an organism respond to drugs, chemical agents, and environmental stressors. Toxicogenomic/genomic biomarker typically defined as differentially expressed genes (DEGs) between chemical-treatment and control groups of samples, and their associated chemical agents are referred to as their regulators. For example, some previous studies reported a set of genes (*MGST2*, *G6PD*, *GSR,* and *GCLC*) as toxicogenomic biomarkers in rat liver since they showed differential expression patterns due to the influence of glutathione-depleting compounds^6–9^. According to biological phenomena, a group of samples might up-regulate one group of DEGs and down-regulate another group of DEGs. As an example, glycolysis and gluconeogenesis pathways-related genes showed upregulation, downregulation, and un-regulation with different clusters of chemical agents, respectively^10^.

In the context of toxicogenomics, separate chemical compound groups exhibit unique toxicity patterns and mechanisms of action toward various DEG groups. This process suggests that the influence of one group of similar chemical agents may upregulate a group of DEG, while this group of chemical agents may downregulate another group of DEG^2,10–12^. Therefore, in toxicogenomic studies, it is important to explore upregulatory, downregulatory, and unregulatory co-clusters between gene clusters and chemical clusters^10–13^. Genes that belong to the upregulatory and/or downregulatory co-clusters are known as toxicogenomic biomarkers, and associated chemical agents belonging to the same co-clusters are known as their regulators^11,14^. These co-clusters are what we refer to as biomarker co-clusters. However, the genes in the unregulatory co-clusters are not regulated by the DCs. Thus, we can infer from this discussion that toxicogenomic data analysis has three primary objectives. Identifying toxicogenomic biomarkers, also known as DEGs, is the first step. Predicting the pattern of gene clusters over DC clusters of similar mechanisms of action and vice versa simultaneously is the second. Third, identification of biomarker (upregulatory and/or downregulatory) co-clusters made up of toxicogenomic biomarkers (DEGs), and their associated chemical regulators. Since DEG identification and cluster pattern prediction of genes and DCs are done simultaneously in objective three, achieving it will mean that the first and second objectives have already been accomplished.

Several statistical techniques, such as the t-test, SAM, LIMMA, ANOVA, and some of their robust approaches, were employed to identify DEGs when comparing chemically treated vs. control or case–control samples to accomplish the first objective^15–19^. Additionally, to identify DEGs from toxicogenomic data, two machine learning-based algorithms exist: the filter and wrapper methods^20^. Comparing the chemically treated and control group of samples of the toxicogenomic data filter method offers a quicker and easier way to discover DEGs^21^. The wrapper method evaluates DEGs according to their capacity to improve the classification models’ accuracy by using feature selection and classification techniques to find the optimal set of DEGs^22,23^. To achieve the second objective of the toxicogenomic data analysis, bi-clustering or co-clustering approaches can be applied to predict the pattern of gene clusters over DC clusters and vice versa simultaneously. Nevertheless, bi-clustering methods only work when row and column clusters are equal and they have no criterion for detecting upregulatory and downregulatory co-clusters^5,13,14,24–28^. However, according to the toxicity mechanism of chemical agents, there are an unequal number of clusters for chemically treated samples and genes in toxicogenomic data^2,11,12,24^. Furthermore, different bi-clustering techniques yield varying numbers of clusters for the same dataset^29^ and cannot predict the significant upregulatory and downregulatory co-clusters which is an important objective of toxicogenomic data analysis. On the contrary, though some traditional hierarchical and model-based clustering techniques can produce an unequal number of latent patterns or clusters of genes or samples^30–37^, these clustering techniques do not, however, provide directions on co-clustering and how to extract important upregulatory and/or downregulatory co-clusters. In this regard, from the perspective of computational burdens and difficulties, the hierarchical co-clustering (HCoClust) approach^11^ is considerably faster, simpler, and more adaptable. It is not, however, robust to anomalous observations. Since there are many steps to generate gene expression datasets are often messed up by observations that are out of the ordinary^38–40^. Toxicogenomic data analysis’s third goal is to identify upregulatory and/or downregulatory co-clusters made up of toxicogenomic biomarkers (DEGs) and the chemical regulators that operate in tandem with them. The HCoClust technique is not capable of doing this. Therefore, to overcome these limitations, in this paper, we proposed a robust hierarchical co-clustering (rHCoClust) approach and its r-package “rhcoclust” for investigating toxicogenomic biomarkers and their associated chemical regulators from the standpoint of robustness.

## Materials and methods

### Toxicogenomic data generation

**Fig. 1:**
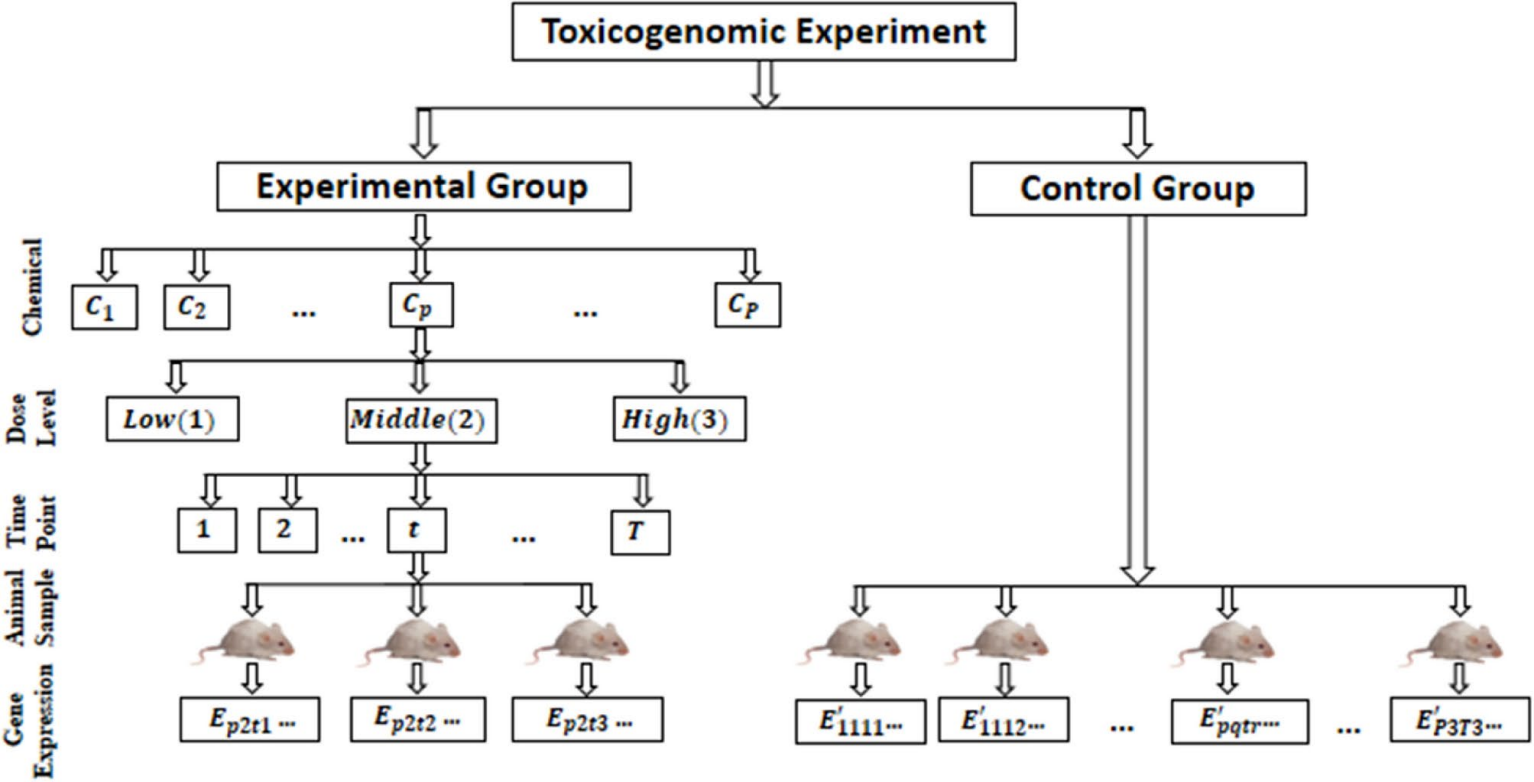
An overview of toxicogenomic data generation processes.

Animal samples from the treatment and control groups often make up a toxicogenomic experiment. The animals in the treatment group get DCs at different intervals of time. Figure 1 demonstrates this experiment. Animal samples from the treatment and control groups are collected, and the gene expression data is generated. Following that, fold change gene expression (FCGE) data are generated from the gene expression of the control and treated groups of animals.

FCGE data are currently used in many toxicogenomic studies because they directly reflect treatment effects on animal samples^5,7,11,12,14^. The FCGE data can be computed using the following equation:

1 $${FC}_{pqtr}={log}_{2}\left(\frac{{E}_{pqtr}}{{E}_{pqtr}^{\prime}}\right)={log}_{2}\left({E}_{pqtr}\right)-{log}_{2}\left({E}_{pqtr}^{\prime}\right)$$

In Eq. (1), ${{\varvec{E}}}_{{\varvec{p}}{\varvec{q}}{\varvec{t}}{\varvec{r}}}$ and ${{\varvec{E}}}_{{\varvec{p}}{\varvec{q}}{\varvec{t}}{\varvec{r}}}^{\boldsymbol{^{\prime}}}$ are the gene expressions for the treatment and control samples respectively and ${{\varvec{F}}{\varvec{C}}}_{{\varvec{p}}{\varvec{q}}{\varvec{t}}{\varvec{r}}}$ is the FCGE for the ${\varvec{p}}^{\varvec{th}}{\varvec{(p}}=\mathbf{1, 2, \ldots,} {\varvec{P)}}$ chemical compound, ${{\varvec{q}}}^{{\varvec{th}}}{\varvec{(q}}=\mathbf{1,2,\ldots} {\varvec{Q)}}$ dose level, ${{\varvec{t}}}^{{\varvec{th}}}{\varvec{(t}}=\mathbf{1,2,\ldots,}{\varvec{T)}}$ time point and ${{\varvec{r}}}^{{\varvec{th}}}{\varvec{(r}}=\mathbf{1,2,\ldots},{\varvec{m=3)}}$ animal sample. Then the toxicogenomic FCGEs data matrix (*F*) can be expressed as:

2 $$F = \left[ {f\overline{{\left( {G_{i} ,DC_{j} } \right)}} .} \right]_{{N \times C}} = [\bar{F}_{{ij}} .]_{{N \times C}}$$

Here ${G}_{i}$ denotes the *i*th gene and ${DC}_{j}$ denotes the *j*th dose of chemical (DC), and $f\overline{{\left( {G_{i} ,DC_{j} } \right)}} .$ = ${\overline{F} }_{ij.}=\frac{1}{m}\sum\nolimits_{r=1}^{m}{F}_{ijr}$ is the mean of $m= 3$ replications, where the dataset {$\bar{F}_{{ij.}} |i = {\text{1,2}}, \ldots ,N;$ $j = {\text{ 1,2}}, \ldots ,$ $C = P \times Q \times T\}$ and $\{ \overline{{FC}} _{{pqt.}} |p = 1,2, \ldots ,P;$ $q = 1,2, \ldots ,Q;$ $t = {\text{1,2}}, \ldots ,T\}$ are equal. According to the statistical control chart (SSC), in the data matrix, gene G_*i*_ is un-regulated by DC_*j*_ if $\text{Lower Control Limit }\left(LCL\right)\le {\overline{F} }_{ij.}\le \text{Upper Control Limit }(UCL)$, significantly upregulated if ${\overline{F} }_{ij.}\ge UCL$, and downregulated if ${\overline{F} }_{ij.}\le LCL,$ while the $\text{central line} (CL)$, $LCL$, and $UCL$ are computed from ${FC}_{pqtr}$ based on the SCC described in section S3 (supplementary material).

### Robust hierarchical co-clustering (rHCoClust) algorithm (proposed)

There are three ways of getting robust results in the presence of outlying observations in the dataset: (i) development of a robust algorithm (ii) applying a classical algorithm after removing outlying observations and (iii) applying a classical algorithm to the transformed dataset^2,41–43^. The last two approaches seem comparatively easier and more flexible than the first approach for robust computation. Nonetheless, deletion of outlying observations loses information from the dataset. Thus, the transformation-based approaches are comparatively popular for robust computation^2,41–43^. Consequently, we considered the logistic transformation of the dataset in this study to reduce outlier effects from hierarchical clustering (HC) and HCoClust. The proposed robustification procedure of HCoClust was designed by incorporating the pre-processing and post-processing steps in HC as follows:

*Step 1* To reduce the influence of outliers in HC and HCoClust, we transformed the FCGE dataset by the logistic function:

3 $$L\left( {G_{i} ,DC_{j} } \right) = \left[ {\frac{1}{{1 + {\text{exp}}\left\{ { - f\overline{{\left( {G_{i} ,DC_{j} } \right)}} .} \right\}}}} \right] = x_{{ij}} ,({\text{say}})$$

where $f\overline{{\left( {G_{i} ,DC_{j} } \right)}} . = \bar{F}_{{ij.}}$ is defined in Eq. (2). If $f\overline{{\left( {G_{i} ,DC_{j} } \right)}} .$ = $\bar{F}_{{ij.}} = 0;$ its transformed value $L\left( {G_{i} ,DC_{j} } \right) = x_{{ij}} = 0.5$ (Eq. 3). Similarly if $f\overline{{\left( {G_{i} ,DC_{j} } \right)}}.$ = $\bar{F}_{{ij.}} > 0; = > 0.5$ < $x_{{ij}} \le 1.0$, and if $f\overline{{\left( {G_{i} ,DC_{j} } \right)}} . = \bar{F}_{{ij.}} < 0; = > 0 \le x_{{ij}} < 0.5$. Therefore, the proposed logistic function (Eq. 3) is bounded within the range of 0.00–1.00 for any FCGE values, and hence, the proposed rHCoClust algorithm produces robust results against outliers.

*Step 2* Construct HC for genes and DCs separately of transformed data matrix [${x}_{ij}]$ from Step 1. Choose the number of gene clusters (GCs) and DC clusters (DCCs) from the dendrograms of HC. Thereafter, divide genes and DCs into the respective GCs and DCCs.

**Fig. 2:**
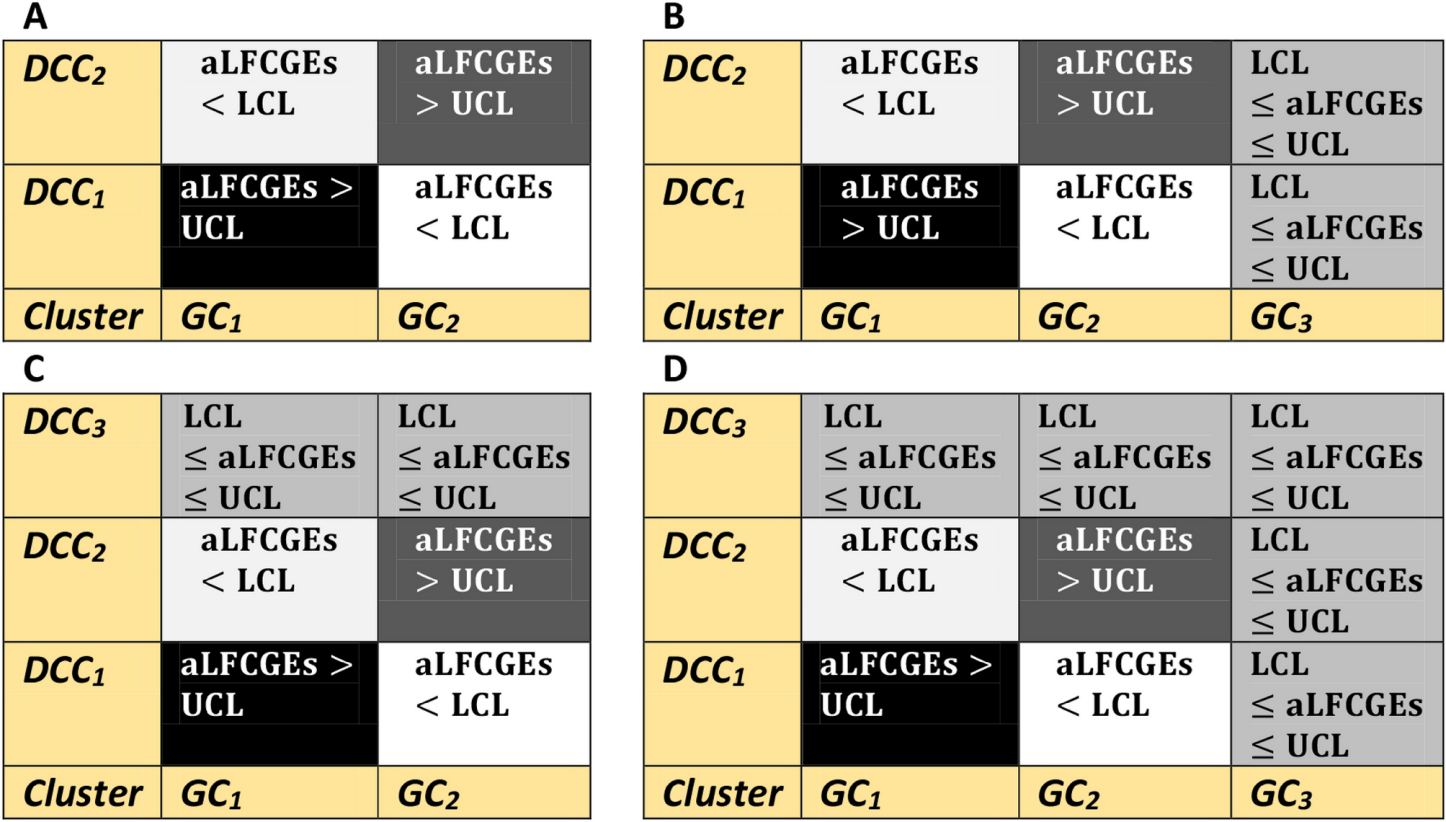
Schematic diagrams of ordered clusters and co-clusters of GCs and DCCs. (**A**) represents two GCs, two DCCs, two upregulatory co-clusters (aLFCGEs > UCL), and two downregulatory co-clusters (aLFCGEs < LCL) that were arranged corresponding to the descending order of upregulatory co-clusters in the principal diagonal. (**B**) represents two GCs, three DCCs, two upregulatory co-clusters (aLFCGEs > UCL), two downregulatory co-clusters (aLFCGEs < LCL), and two unregulatory (LCL ≤ aLFCGEs ≤ UCL) co-clusters corresponding to the GC_3_ that were arranged according to the descending order of upregulatory co-cluster diagonally. (**C**) represents three GCs, two DCCs, two upregulatory co-clusters (aLFCGEs > UCL), two downregulatory co-clusters (aLFCGEs < LCL), and two unregulated (LCL ≤ aLFCGEs ≤ UCL) co-clusters corresponding to the DCC_3_ were arranged according to the descending order of the upregulatory co-clusters diagonally. (**D**) Represents three GCs, three DCCs, two upregulatory co-clusters (aLFCGEs > UCL), two downregulatory co-clusters (aLFCGEs < LCL), five unregulatory (LCL ≤ aLFCGEs ≤ UCL) co-clusters corresponding to the GC_3_ and DCC_3_ arranged corresponding to the descending order of upregulatory co-clusters in the principal diagonal.

*Step 3* Each combination of DCCs and GCs forms a co-cluster. Compute the average of logistically transformed FCGEs (aLFCGEs) of all coordinates within a co-cluster. Similarly, compute aLFCGEs for the other co-clusters. Then order the GCs on the X-axis and the DCCs on the Y-axis, corresponding to the descending order of aLFCGEs for upregulatory co-clusters (Fig. 2). The co-cluster (GC_1_, DCC_1_) is said to be the top-ranked upregulatory co-cluster in panels A, B, C, and D of Fig. 2. Similarly, the co-cluster (GC_2_, DCC_2_) is the second-top-ranked upregulatory co-cluster in all panels.

*Step 4* Significant co-clusters in the logistically transformed data matrix can be determined using the SCC. The significant upregulatory and downregulatory co-clusters can be found using the UCL and LCL of SCC (Supplementary Section S3). A co-cluster is said to be upregulatory if aLFCGEs > UCL and downregulatory if aLFCGEs < LCL. Otherwise, it is referred to as an unregulatory co-cluster^2,44,45^. A few basic schematic representations of co-clusters that are either upregulatory, downregulatory, or unregulatory are shown in Fig. 2. The SCC is useful in separating assignable causes of variation from random or natural causes of variation in a production process, we considered it in our current situation^2,44,45^.

### Robustness of the proposed method (rHCoClust)

We investigated the robustness and cluster stability of rHCoClust over the classical methods HC and HCoClust by using the clustering error rate (ER)^14^ and Tukey’s sensitivity curve (SC)^46,47^ based on simulated data. Evaluation measures: accuracy, sensitivity, and specificity based on real datasets are also used to assess the robustness and cluster stability of rHCoClust over HC and HCoClust.

#### Error rate (ER)

In the absence or presence of outlying observations in the data, the clustering ER^14^ was estimated. Miss-clustered observations are those where the genes or DCs in the clusters were incorrectly assigned by using the proposed or conventional approach. The clustering ER represents the percentage of miss clustered observatories that can be mathematically expressed as:

$$\left(\frac{\text{Tolal number of missclustered observations}}{\text{Total number observations}}\right)\times 100$$

#### Sensitivity curve (SC)

The influence of outliers in the proposed procedure can be measured by SC^46,47^ as follows:

4 $${SC}_{n}\left({y}^{*}\right)=n\left[T\left({y}_{1,}{y}_{2},\dots ,{y}_{n-1},{y}^{*}\right)-T\left({y}_{1,}{y}_{2},\dots ,{y}_{n-1}\right)\right],$$

Let ${T(F}_{n})=T\left({y}_{1,}{y}_{2},\dots ,{y}_{n}\right)=\sum_{j=1}^{n}{|y}_{ij}-{y}_{kj}|$ represent the 1-norm distance between the logistically transformed FCGEs of two genes G_*i*_ and G_*k*_, where *y*_*ij*_ is defined in Eq. (3), *F*_*n*_ is the empirical distribution of logistically transformed FCGEs ${y}_{1,}{y}_{2},\dots ,{y}_{n}$ of two genes, then the sensitivity curve reduces to.

5 $$SC_{n} \left( {y^{*} } \right) = \frac{{\left[ {T\left( {\left( {1 - \frac{1}{n}} \right)F_{n} + \frac{1}{n}\Delta_{{y{*}}} } \right) - T\left( {F_{n - 1} } \right)} \right]}}{\frac{1}{n}},$$

where ${\Delta }_{y*}$ is the likelihood measure that positions mass 1 at the position y*. The sensitivity curve ${SC}_{n}\left({y}^{*}\right)$ converges to the influence function for n → ∞, as follows.

6 $$IF\left( {y^{*} ;T, F} \right) = - T\left( F \right) + |y_{i}^{*} - y_{k}^{*}| ,$$

which is finite, since ${|y}_{i}^{*}-{y}_{k}^{*}|$ is finite for any FC values and $T\left(F\right)$ represents the actual distance between *G*_*i*_ and *G*_*k*_ genes. Thus, the proposed estimator T of distance is said to be robust under the distribution F.

#### Cluster evaluation metric

We employed the accuracy, sensitivity, and specificity evaluation metrics based on machine learning (ML) techniques to assess the cluster stability and performance between the proposed rHCoClust and HCoClust approaches to the real data aspect and when it contaminated with 5% outliers based on Tukey-Huber contamination model (THCM)^14,48^. In this context, we examined the rat sample dataset treated with three dose levels (low, middle, and high) of glutathione-depleting compounds (acetaminophen, methapyrilene, and nitrofurazone)^7^ and PPARs-related gene regulatory compounds (WY-14643, clofibrate, gemfibrozil, benzbromarone, and aspirin)^48^ after four time periods. Therefore, it was simpler to predict gold standard patterns or clusters in the chemically treated samples and the genes since the toxicity mechanism of these compounds on the genes involved in the pathway is known. Following our examination of the dataset’s dendrogram for the row (gene) and column (DCs) entities, we forecast three DC clusters and five gene clusters. Treating these clusters as gold standard examples, we trained random forest (RF)^49,50^, support vector machine (SVM)^51,52^, and gradient boosting machine (GBM)^53,54^ ML models were used to predict the accuracy, sensitivity, and specificity of these clusters in the absence and presence of 5% outliers in the same dataset. These ML models were trained using three iterations of tenfold cross-validation (CV) and the test dataset and then employed to compute specificity, sensitivity, and accuracy.

### Networking of co-clusters

In biological phenomena, a group of genes is usually involved in performing a specific biological function and responding to a specific set of exposures (DCs). Consequently, groups of genes (GCs) interact with their regulatory groups of DCs (DCCs) via the co-clusters^5,10,11,14^. Therefore, the GCs and DCCs make networks themselves, and genes and DCs in the GCs and DCCs make networks through the co-clusters^2^. The rHCoClust algorithm performed this job by using two types of networking: (1) GCs and DCCs networking; and (2) genes and DCs networking through co-clusters. The edge thickness of the GCs and DCCs networks represents corresponding aLFCGEs values. The red and green edges represent significant upregulatory and downregulatory relationships, otherwise, the relationship is insignificant (Fig. 6 and Supplementary Figs. S8, S9, and S12).

### Simulated dataset

We used a simulated dataset to compare how well the proposed (rHCoClust) and traditional (HCoClust) methods worked. This comparison was done based on the distance and HC method combinations (Euclidean: Ward.D, Euclidean: Ward.D2, Manhattan: Ward.D, Manhattan: Ward.D2, Minkowski: Ward.D and Minkowski: Ward.D2) in the absence and presence of outlying observations. We choose these combinations since HCoClust co-cluster genes and DCs in combination with the mentioned distance and “Ward’s” agglomerative clustering methods^11^. However, the proposed rHCoClust approach and its r-package “rhcoclust” are flexible in choosing the distance and agglomerative clustering methods. Consequently, we generated pathway-level FCGE data of size $\left(N=50\times C=36\right)$ at the 24-h time point, since toxicity effects were more apparent at this time point^7^. Due to exposure to a homogenous set of DCCs, certain genes are up-regulated, and some are down-regulated, which is an important characteristic of toxicogenomic gene expression data. According to this nature, we simulated FCGE data $({FC}_{pqr})$ using the following model:

7 $$FC_{pqr} = \begin{array}{*{20}c} {{\varvec{GC}}_{4} } & {0.00} & {0.00} & {0.00} \\ {{\varvec{GC}}_{3} } & {0.00} & { - {3}.00} & {0.00} \\ {{\varvec{GC}}_{2} } & { - {3}.00} & { + {3}.00} & {0.00} \\ {{\varvec{GC}}_{1} } & { + {3}.00} & {0.00} & {0.00} \\ {{\varvec{Clusters}}} & {{\varvec{DCC}}_{1} } & {{\varvec{DCC}}_{2} } & {{\varvec{DCC}}_{3} } \\ \end{array} + N\left( {0, 0.35} \right)$$

The simulated dataset contains three DC groups/clusters (DCCs) and four gene groups/clusters (GCs). Genes G1-G10, G11-G20, G21-G30, and G31-G50, respectively, made up GC 1, 2, 3, and 4. The DCs C1 High-C5 High-C1 Middle-C5 Middle, C6 High-C10 High-C6 Middle-C10 Middle, and C1 Low-C12 Low-C11 Middle-C12 Middle-C11 High-C12 High, respectively, made up the DCC 1, 2, and 3. Where G stands for gene and DC stands for chemical doses, which were organized along the row and column, respectively in the simulated data matrix. Each component of the simulated dataset added the error term N(0, 0.35) from a normal distribution with a mean of 0 and variance of 0.35. The simulated dataset demonstrated that the DCC1 is responsible for upregulating GC1, whereas the DCC2 and DCC1 are responsible for up- and down-regulating GC2. DCC2 downregulates GC3. None of the DCCs regulate the GC4, and neither does DCC3 expose any of the genes in the pathway dataset. To examine the performance of the suggested rHCoClust algorithm, we contaminated the simulated dataset with outliers following the Tukey-Huber contamination model (THCM)^14,48^ and the independent contamination model (ICM)^14,55^. The Supplemental Section S2 provided details of the THCM and ICM data contamination techniques.

### Real datasets

We used three sets of pathway-level gene expression data from the Japanese Toxicogenomics Project (TGP)^56^ to compare the performance and cluster stability of the suggested rHCoClust and HCoClust algorithms using accuracy, sensitivity, and specificity based on ML methods and to forecast the suitability of the rHCoClust approach for practical use. In the first dataset, FCGE data were acquired from the rat samples that were treated with three dose levels (low, middle, and high) of glutathione-depleting compounds (acetaminophen, methapyrilene, and nitrofurazone)^7^ and PPARs-related gene regulatory compounds (WY-14643, clofibrate, gemfibrozil, benzbromarone, and aspirin)^57^ after four time periods. We named this dataset GMP-PPAR and used it to evaluate the performance and cluster stability of the proposed rHCoClust algorithm in comparison to HCoClust, as well as to assess the applicability of rHCoClust for analyzing real-world toxicogenomic data. The remaining datasets, referred to as the GMP and PPAR-SP datasets, respectively, were obtained using FCGE data for the glutathione metabolism pathway (GMP) and the PPAR signaling pathway (PPAR-SP). We took into account all dose levels (Low, Middle, and High) for three glutathione-depleting compounds (acetaminophen, methapyrilene, and nitrofurazone) and seven non-glutathione-depleting compounds (erythromycin, hexachlorobenzene, isoniazid, gentamicin, glibenclamide, penicillamine, and perhexiline)^7^ in the GMP dataset. In contrast, for the PPAR-SP dataset, we considered PPARs-related gene regulatory compounds (WY-14643, clofibrate, gemfibrozil, benzbromarone, and aspirin)^57^ as well as a few other randomly chosen compounds (cisplatin, diltiazem, methapyrilene, phenobarbital, and triazolam), along with their dose levels (low, middle, and high) at multiple time points. We obtained FCGE data for the GMP-PPAR, GMP, and PPAR-SP datasets from “Toxygates” (https://toxygates.nibiohn.go.jp/toxygates/#columns)^7^ which is an online toxicogenomic database and analysis platform. To show the clear difference in the performance between rHCoClust and HCoClust with the real dataset, we contaminated 5% of data in the GMP-PPAR dataset with outliers following the Tukey-Huber contamination model (THCM)^14,48^.

### Ethical clearance

The study did not require ethics clearance because, as previously stated, the data were sourced from publicly accessible sources.

## Results

### Simulation study

**Fig. 3:**
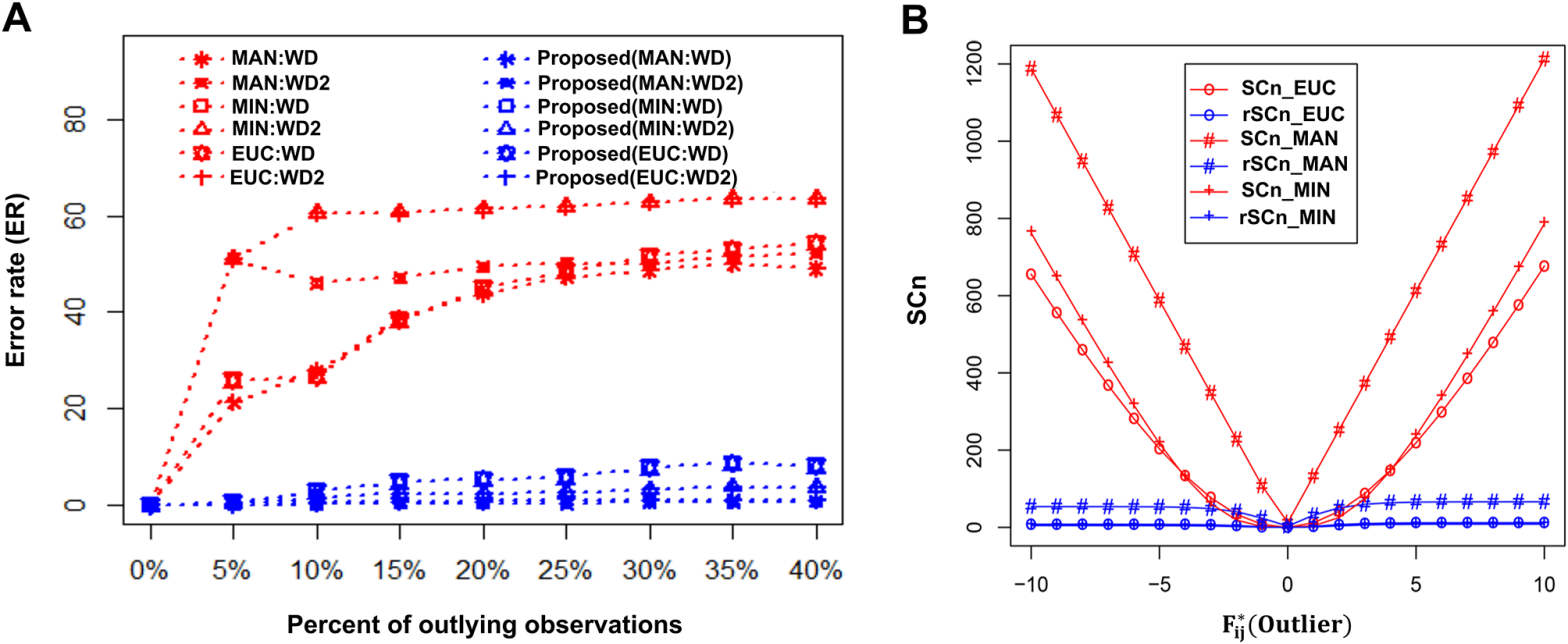
Performance investigation using simulated data of the proposed rHCoClust over the conventional HCoClust. (**A**) Clustering ER of genes in the absence and presence of different ranges (0–40%) of THCM-based outliers. (**B**) Sensitivity curves for the proposed and conventional methods over a range of outliers (− 10 to + 10). In the figure SCn = classical method, rSCn = proposed method, MAN = manhattan, MIN = minkowski, EUC = Euclidean, WD = Words’method1 and WD2 = Words’method2.

As previously stated, HCoClust is faster, simpler, and adaptable for clustering and co-clustering the toxicogenomic data based on the Euclidean:Ward, Manhattan:Ward, and Minkowski:Ward distance and HC method combinations^11^. However, these approaches are extremely vulnerable to outlier observations in the dataset. We evaluated the efficiency of the proposed rHCoClust and HCoClust algorithms for the distance and agglomerative HC method combinations (Manhattan: Ward.D, Minkowski: Ward.D2, and Euclidean: Ward.D). As there are two variations on the “Ward’s” method^58^ used in the “hclust” function of the “stats” package in R, we use “Ward.D” and “Ward.D2” instead of the “Ward’s” method. The descriptions of the distance and agglomerative HC algorithms were given in supplementary material (Section S1: S1.1 and S1.2). In the simulated dataset Fig. S1A, there were four clusters in the row entity (gene) (Fig. S1C) and three clusters in the column entity (DC) (Fig. S1D) retrieved from a random mixture of simulated data (Fig. S1B). Tukey’s SC and clustering ER were used to examine the performance of the rHCoClust algorithm in comparison to HCoClust in simulated dataset. In the case of ER calculation, the simulated data were contaminated over the ranges (THCM: 0–40%, ICM: 0.00–0.598) of outlying observations. Similarly, Tukey’s SC curve was generated by introducing weights (− 10 to + 10) on specific observations. Figures 3 and S3 compared the performance of the proposed and classical approaches. From these figures, it is observed that rHCoClust approach outperforms the classical approach in the presence of outliers in the dataset. Otherwise, they perform equally (0% ER) without outliers in the dataset (Figs. 3 and S3). Therefore, the proposed algorithm is also less sensitive to outlying observations. Figure S4 visualized the original data structure, row and column entities randomly allocating data structure, and methods (HCoClust and rHCoClust) reconstructed data structure in the absence and presence of outliers (10%) in the simulated dataset. From this figure, it is observed that the proposed approach reconstructs the data structure more efficiently in the absence and presence of outliers. These results proved that the proposed rHCoClust algorithm is far better than the classical HCoClust algorithm in clustering and co-clustering genes and DCs in the presence and absence of outliers in the dataset. Even when there are missing observations in the dataset, the suggested rHCoClust algorithm is effective for clustering and co-clustering. In that circumstance, any observation, even outlier observations, can replace any missing observations. The performance of the rHCoClust algorithm was also evaluated in comparison to the conventional bi-clustering methods (BCBimax^26^, BCCC^27^, BCPlaid^59^, BCQuest^60^, BCQuestmet^60^, BCQuesttord^60^, and BCSpectral^61^) using simulated data. The nature of toxicogenomic data is that a cluster of DCC forms a co-cluster with each cluster of GCs. Thus, in the simulated data (Fig. S1A/S4A and Fig. S13 Simulated Data), four GCs (Fig. S1C) and three DCCs (Fig. S1D) created twelve co-clusters. Figure S13(rHCoClust) demonstrated how accurately rHCoClust recovered co-clusters from the mixed data (Fig. S13 Mixed Data) compared to the conventional bi-clustering methods. The proposed rHCoClust correctly identified the two upregulatory and two downregulatory co-clusters (Fig. S13rHCoClust, Table S7, and Table 2), which the conventional bi-clustering approaches cannot reconstruct accurately (Fig. S13). Nevertheless, though there are twelve co-clusters in the simulated data identified by rHCoClust (Figures S6 and S9, Table S7), the bi-clustering techniques, BCBimax, BCCC, BCPlaid, BCQuest, BCQuestmet, BCQuesttord, and BCSpectral, produced 3, 2, 5, 0, 3, 1, and 2 co-clusters, respectively (Table S7). As a result, traditional bi-clustering methods can’t retrieve the hidden patterns in simulated data like the rHCoClust can. The ranked aLFCGEs within co-clusters and their associated GCs and DCCs along with their newly assigned cluster numbers, were given in Table 2. Then, we used SCC on the aLFCGE values made by the rHCoClust algorithm for the co-clusters to find biomarker co-clusters to investigate the DEGs and their regulatory DCs. Figure S6 and Table 2 showed the biomarker co-clusters and the GCs and DCCs that went with them for the simulated data. In the supplementary material (Figs. S8 and S9), we showed the GCs and DCCs network and the gene-DC networks through the co-clusters, respectively, for simulated data.

### Real data analysis

To compare the performance and cluster stability between rHCoClust and HCoClust approaches in the real-life data aspect, at first the GMP-PPAR dataset was used. The ML techniques of RF, SVM, and GBM were applied to calculate the evaluation metrics: accuracy, sensitivity, and specificity to compare the clustering stability and performance of the proposed rHCoClust over the HCoClust algorithm. In this regard, in the GMP-PPAR dataset, we consider the five clusters in the gene and the three clusters in the DCs as the gold standard examples for evaluation, as stated in Section “Robustness of the proposed method (rHCoClust)” (cluster evaluation metric). Consequently, we saw that rHCoClust produced higher accuracy, sensitivity, and specificity for all considered ML methods RF, SVM, and GBM (Table 1). However, if we modify 5% of data with THCM-based outliers^14,48^, rHCoClust shows considerably better results compared to the HCoClust (Table 1). Thus, the suggested rHCoClust algorithm performed better or produced a more stable cluster and co-cluster than the HCoClust algorithm in the real dataset scenario. The GMP-PPAR dataset’s co-cluster view, biomarker co-cluster in concurrence with gene and DC clusters, and gene-DC network along with significant gene-DC network processed by the rHCoClust algorithm were displayed, respectively, in Supplemental Figs. S10, S11, and S12. Additionally, supplemental Table S2 gives the gene and DC cluster numbers of this real (GMP-PPAR) dataset, their ranked co-cluster mean, and significant upregulatory and downregulatory biomarker co-clusters generated by the rHCoClust algorithm. Table S3 depicts the gene and DC clusters for the GMP-PPAR dataset.

**Table 1:** The average ± SD values of accuracy, sensitivity, and specificity for ML approaches (RF, SVM, and GBM) based on tenfold CV on the clustering results of rHCoClust (HCoClust) for the GMP-PPAR dataset.

ML methods	Classification of clustered Genes in the absence of outliers			Classification of clustered Genes in the presence of 5% THCM-based outliers		
	Accuracy	Sensitivity	Specificity	Accuracy	Sensitivity	Specificity
RF	0.855 ± 0.022 (0.852 ± 0.032)	0.782 ± 0.105 (0.774 ± 0.141)	0.958 ± 0.019 (0.957 ± 0.019)	0.832 ± 0.088 (0.742 ± 0.092)	0.752 ± 0.723 (0.674 ± 0.841)	0.938 ± 0.098 (0.837 ± 0.103)
SVM	0.706 ± 0.033 (0.687 ± 0.049)	0.727 ± 0.019 (0.699 ± 0.036)	0.912 ± 0.019 (0.700 ± 0.035)	0.68 ± 0.072 (0.617 ± 0.096)	0.695 ± 0.089 (0.609 ± 0.092)	0.882 ± 0.099 (0.660 ± 0.115)
GBM	0.858 ± 0.037 (0.847 ± 0.040)	0.843 ± 0.112 (0.829 ± 0.154)	0.961 ± 0.028 (0.957 ± 0.028)	0.828 ± 0.074 (0.737 ± 0.073)	0.802 ± 0.132 (0.765 ± 0.144)	0.921 ± 0.074 (0.854 ± 0.083)

ML methods	Classification of clustered DCs in the absence of outliers			Classification of clustered DCs in the presence of 5% THCM-based outliers		
	Accuracy	Sensitivity	Specificity	Accuracy	Sensitivity	Specificity
RF	0.954 ± 0.028 (0.952 ± 0.052)	0.951 ± 0.051 (0.948 ± 0.072)	0.972 ± 0.022 (0.972 ± 0.022)	0.922 ± 0.087 (0.812 ± 0.094)	0.931 ± 0.044 (0.868 ± 0.072)	0.942 ± 0.082 (0.822 ± 0.092)
SVM	0.956 ± 0.005 (0.953 ± 0.008)	0.961 ± 0.009 (0.959 ± 0.009)	0.974 ± 0.003 (0.972 ± 0.022)	0.923 ± 0.084 (0.803 ± 0.115)	0.925 ± 0.081 (0.841 ± 0.091)	0.943 ± 0.092 (0.872 ± 0.098)
GBM	0.947 ± 0.023 (0.944 ± 0.023)	0.948 ± 0.032 (0.947 ± 0.038)	0.970 ± 0.009 (0.968 ± 0.010)	0.917 ± 0.083 (0.834 ± 0.153)	0.901 ± 0.094 (0.803 ± 0.096)	0.920 ± 0.063 (0.821 ± 0.084)

**Fig. 4:**
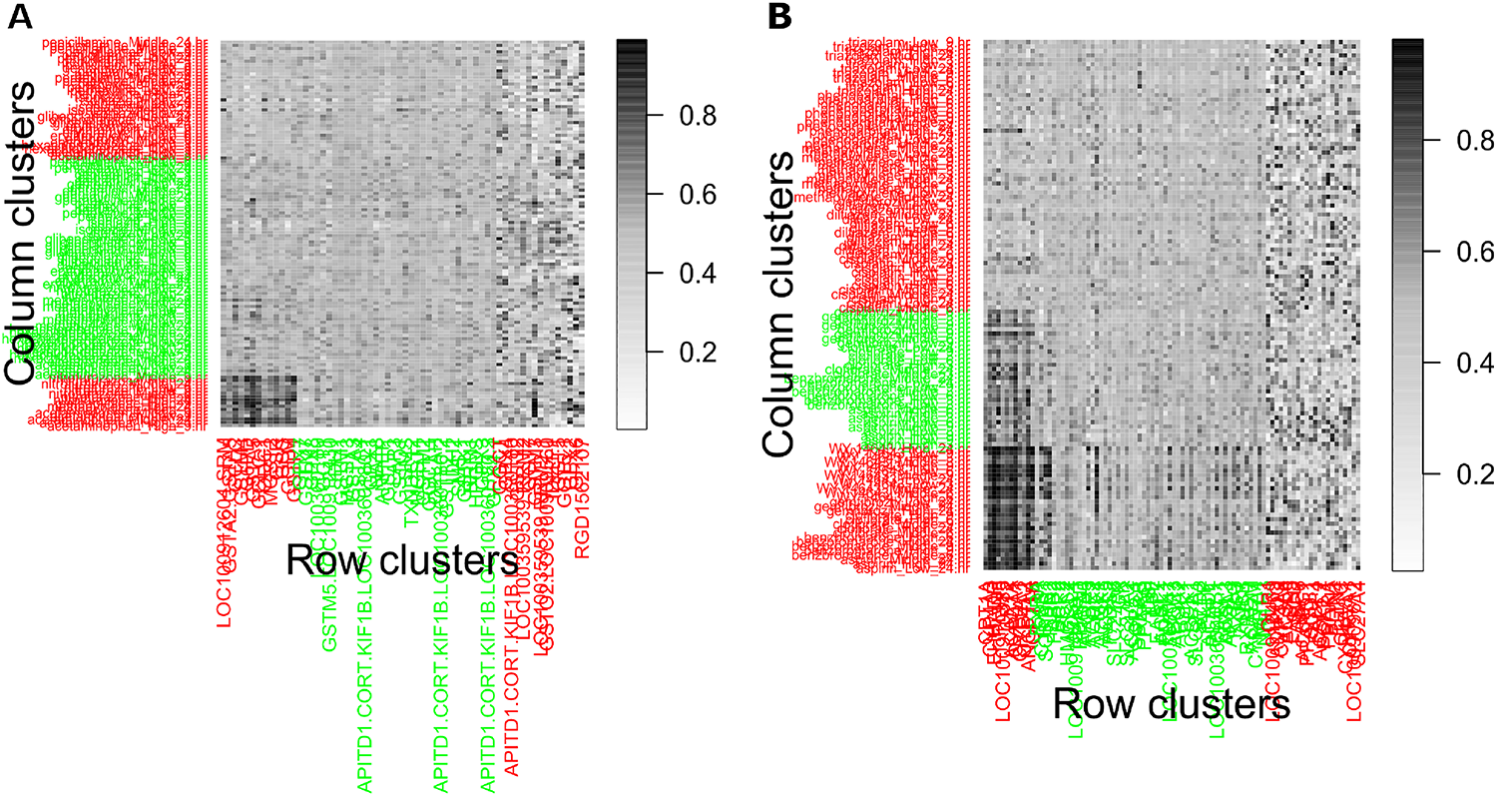
Co-clustered data structure recovered by the proposed rHCoClust method. (**A**) GMP dataset and (**B**) PPAR-SP dataset.

**Fig. 5:**
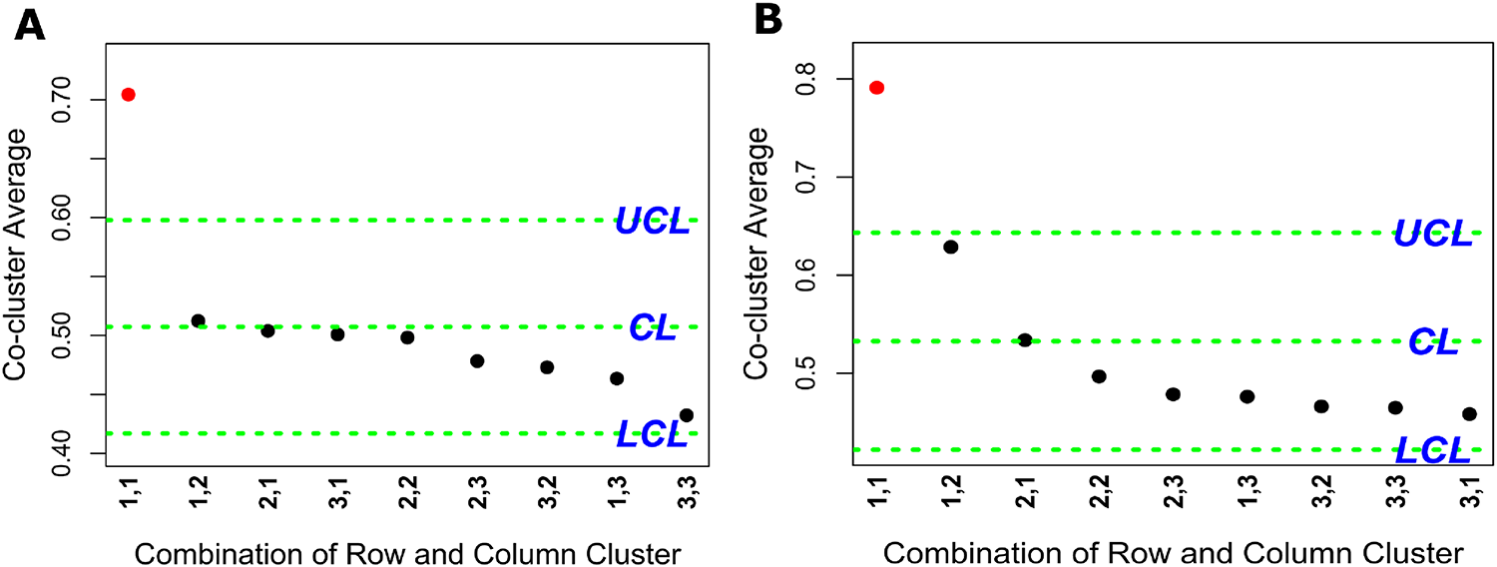
Statistical control chart (SCC) for up-and downregulatory biomarker co-cluster identification. The co-clusters (a combination of GC and DCC makes a co-cluster) refer to plots beyond the UCL or LCL that are considered biomarker co-clusters. (**A**) SCC for the GMP dataset and (**B**) SCC for the PPAR-SP dataset. Accordingly, in Figure (**A**) and (**B**) number of GC and DCC combinations (1,1) are the upregulatory co-clusters for the GMP and the PPAR-SP datasets, respectively.

**Fig. 6:**
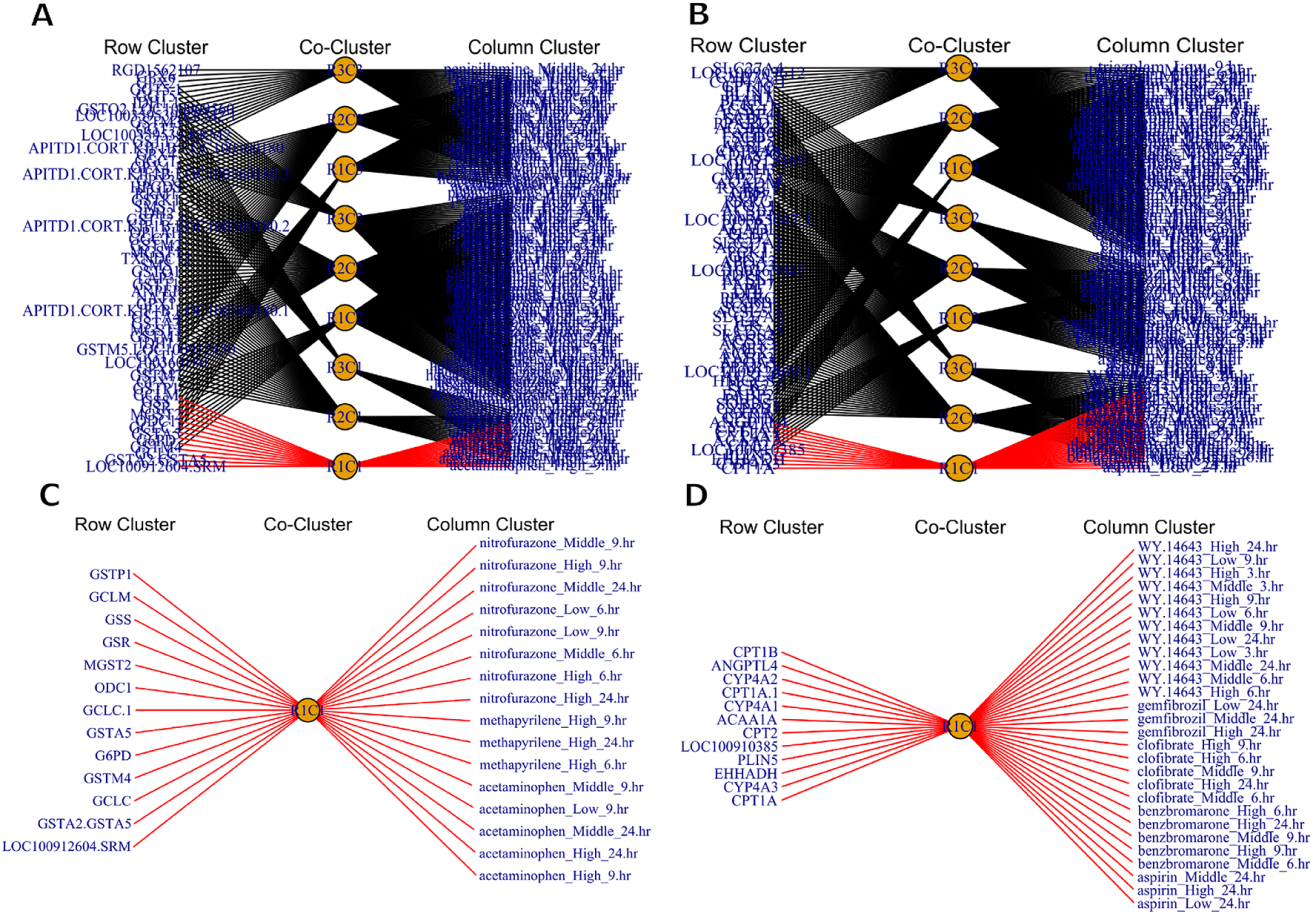
Co-cluster networks between members of row clusters (GCs) and column clusters (DCCs) with black edges indicate insignificant (unregulatory) co-cluster networks, co-cluster networks between members of GCs and DCCs with red edges indicate significant upregulatory co-cluster networks and co-cluster networks between members of GCs and DCCs with blue edges indicate significant downregulatory co-cluster networks. (**A**) Co-cluster networks of the GMP dataset, (**B**) Co-cluster networks of the PPAR-SP dataset, (**C**) Upregulatory (red edges) co-cluster networks separated from (**A**), and (**D**) Upregulatory (red edges) co-cluster networks separated from (**B**).

Two other datasets, GMP and PPAR-SP, were also examined to evaluate the applicability of the rHCoClust approach in toxicogenomic data analysis. Accordingly, the dendrograms for the gene and DC clusters of the GMP and PPAR-SP datasets are shown in Fig. S2. These dendrograms led us to conclude that each of the GMP and PPAR-SP datasets contains three GCs and three DCCs. Table 2 shows the GC and DCC numbers according to the descending order of aLFCGE values along with significant upregulatory biomarker co-clusters for the GMP and PPAR-SP datasets. Tables S4 and S5 showed the GCs and DCCs generated by the rHCoClust algorithm for the GMP and PPAR-SP datasets, respectively. Figure 4 shown rHCoClust method generated co-cluster view for the real datasets (GMP and PPAR-SP). The biomarker co-clusters together with their associated GC and DCC combinations discovered by the SCC based on the rHCoClust algorithm’s output are shown in Fig. 5. The gene-DC network for the GMP and PPAR-SP datasets and their biomarker co-clusters are shown in Fig. 6 and the GCs and DCCs network for the same datasets are included in the supplemental material (Fig. S7). The DEGs identified from biomarker co-clusters for the GMP and PPAR-SP datasets were functionally annotated with DAVID^62^, and it was discovered that they were highly significant in the associated KEGG^63^ pathways. The results are shown in Table 3 (GMP data) and Table S6 (PPAR-SP data).

**Table 2:** The gene and DC cluster numbers for the simulated and real datasets (GMP and PPAR-SP) produced by the rHCoClust algorithm, together with the ranking of co-cluster means.

Simulated data			GMP data			PPAR-SP data		
GCs-DCCs#	aLFCGEs	Co-cluster significance	GCs-DCCs#	aLFCGEs	Co-cluster significance	GCs-DCCs#	aLFCGEs	Co-cluster significance
**1,1**	**0.94912**	**Sig Up-Reg**	**1,1**	**0.70434**	**Sig Up-Reg**	**1,1**	**0.79117**	**Sig Up-Reg**
**2,2**	**0.94910**	**Sig Up-Reg**	1,2	0.51229	Insignificance	1,2	0.62867	Insignificance
1,3	0.50726	Insignificance	2,1	0.50387	Insignificance	2,1	0.53373	Insignificance
3,1	0.50208	Insignificance	3,1	0.50084	Insignificance	2,2	0.49663	Insignificance
4,3	0.49917	Insignificance	2,2	0.49819	Insignificance	2,3	0.47837	Insignificance
2,3	0.49911	Insignificance	2,3	0.47828	Insignificance	1,3	0.47601	Insignificance
3,3	0.49849	Insignificance	3,2	0.47299	Insignificance	3,2	0.46600	Insignificance
4,2	0.49680	Insignificance	1,3	0.46344	Insignificance	3,3	0.46470	Insignificance
2,1	0.49570	Insignificance	3,3	0.43229	Insignificance	3,1	0.45823	Insignificance
3,2	0.49501	Insignificance						
**1,2**	**0.04833**	**Sig Down-Reg**						
**4,1**	**0.04787**	**Sig Down-Reg**						

Significant upregulatory co-clusters (Sig Up-Reg) and Significant downregulatory co-clusters (Sig Down-Reg).

**Table 3:** Functional annotation of the KEGG pathway on the biomarker genes/DEGs (genes in cluster 1) explored by the rHCoClust algorithm for GMP dataset.

Term (KEGG pathway)	Count	%	*p*-value	Genes
rno00480: Glutathione metabolism	11	91.66	2.43E-22	*MGST2, G6PD, GCLM, ODC1, GSR, GSTA5, GCLC, GSTA2, GSTP1, GSS, GSTM4*
rno00980: Metabolism of xenobiotics by cytochrome	5	41.66	1.23E-6	*MGST2, GSTA5, GSTA2, GSTP1, GSTM4*
rno00982: Drug metabolism—cytochrome	5	41.66	1.30E-6	*MGST2, GSTA5, GSTA2, GSTP1, GSTM4*
rno05204: Chemical carcinogenesis	5	41.66	3.54E-6	*MGST2, GSTA5, GSTA2, GSTP1, GSTM4*
rno01100: Metabolic pathways	5	41.66	0.068	*G6PD, GCLM, ODC1, GCLC, GSS*

## Discussion

Toxicogenomic studies are concerned with separating safe and toxic drugs or chemicals based on genomic biomarkers. This task is an imperative part of toxicology and the drug discovery and development process. The co-clustering approaches are now widely used in toxicogenomic studies to explore upregulatory and downregulatory co-clusters between DEGs and DCs. Each of the upregulated and downregulated DEGs-clusters is influenced by a specific DCC to perform a specific biological task through the respective biological process and pathway^10–14^. The HCoClust algorithm^11^, which is quicker, simpler, and more versatile than other approaches, can be used to do these tasks. HCoClust, however, is not resistant to anomalous observations and can’t predict significant upregulatory and downregulatory biomarker co-clusters. Nonetheless, due to the multiple stages of data production, gene expression databases are frequently tainted by outliers^38–40^. Therefore, in this study, we suggested a more reliable co-clustering method rHCoClust by incorporating pre- and post-processing stages into the HCoClust method. In the case of the pre-processing step (see step 1 in Section “Robust hierarchical co-clustering (rHCoClust) algorithm (proposed)”), we transformed the fold change gene expression (FCGE) data by the logistic function, which leads to robust estimations. Clustering ER and Tukey’s SC were used to examine the robustness property or clustering stability of our estimation for simulated data, while accuracy, sensitivity, and specificity were used for real (GMP-PPAR) data. In the post-processing step (see steps 2–4 in Section “Robust hierarchical co-clustering (rHCoClust) algorithm (proposed)”), we computed aLFCGEs values for all co-clusters and considered the top upregulatory and downregulatory co-clusters, which corresponded to the largest and smallest values of aLFCGEs, respectively. We selected the statistically significant upregulatory and downregulatory co-clusters by using the statistical control chart (SCC). The criteria UCL < aLFCGEs and LCL > aLFCGEs were used to identify statistically significant upregulatory and downregulatory co-clusters respectively.

The performance of the proposed rHCoClust over the conventional HCoClust was investigated using simulated and real data analysis. The simulation study showed the clustering ER of the rHCoClust approach was far smaller than that of conventional HCoClust and rHCoClust also less sensitive to outlying observations observed from the SC. This result was persistent when outliers were incorporated into the simulated dataset using the THCM^48^ and ICM^55^. The, rHCoClust also reconstructs the original structure of the simulated dataset more accurately than HCoClust in the presence of outliers. The conventional HCoClust and the proposed rHCoClust showed almost equal performance only when there were no outlying observations in the dataset. Additionally, rHCoClust outperformed the bi-clustering approaches in detecting co-clusters, since bi-clustering methods only work when row (gene) and column (DC) clusters are equal, and they have no criterion for detecting upregulatory and downregulatory co-clusters. In contrast, the evaluation metrics of accuracy, sensitivity, and specificity based on the ML approach (RF, SVM, and GBM) for the GMP-PPAR dataset confirm that the clustering performance or stability of the proposed rHCoClust is better than that of HCoClust. In the GMP-PPAR dataset acetaminophen, methapyrilene, and nitrofurazone were identified as glutathione-depleting compounds, whereas WY-14643, clofibrate, gemfibrozil, benzbromarone, and aspirin were PPAR-related gene regulating chemicals. When acetaminophen was consumed, there was a significant decrease in liver glutathione and a corresponding observation of centrilobular hepatocyte necrosis^64,65^. Glutathione removes nitrofurazone from the rat liver and high doses of it cause hepatocyte and adrenal necrosis^66,67^. Methapyrilene increases glutathione in the liver and causes fetotoxicity and hepatocyte hypertrophy. Methapyrilene also regulated DEGs that were significantly enriched in terms of “glutathione metabolism,” “apoptosis,” “MAPK signaling pathway,” and “regulation of cell cycle,” all of which were believed to be involved in the development of liver hypertrophy^68,69^. By contrast, treatment with Wy-14643 primarily stimulates hepatic PPARα and hepatocyte hypolipidemia^70^. Clofibrate treatment reduces the severity of hepatocellular necrosis, selective protein arylation caused by acetaminophen, and glutathione depletion in addition to activating PPAR-α in the liver and adipose tissue^71,72^. One of the most often given fibrate anti-dyslipidemia medications is gemfibrozil, a PPAR-α receptor ligand that alters liver function and induces cholestatic jaundice and cholelithiasis^73^. Benzbromarone binds to PPARs in hepatocytes to stimulate the proliferation of peroxisomes and regulates the expression of proteins involved in lipid metabolism^74^. By regulating the PPAR δ-AMPK-PGC-1 α pathway in dyslipidemic conditions, aspirin, and PPAR-α activators improve nonalcoholic fatty liver disease and atherosclerosis. In addition, they suppress monocyte chemoattractant protein-1 production in human endothelial cells that is triggered by high glucose concentration, and high dose of it considered as hepatotoxic agent^75–77^. Consequently, GMP and the PPAR-SP associated genes were taken into consideration in the dataset GMP-PPAR, which is made up of two groups of compounds with two comparable mechanisms of action: glutathione depletion and PPAR-related gene regulatory features. Finally, gold standard clusters could be created using dendrograms based on the genes and DCs that were known to exhibit certain patterns. The suggested rHCoClust might then be compared to HCoClust to examine clustering performance or stability in case real-life data based on assessment metrics accuracy, sensitivity, and specificity considering gold standard clusters as an example. The rHCoClust outperformed HCoClust in the case of real data as well since it produced larger average values for evaluation metrics accuracy, sensitivity, and specificity (Table 1). Furthermore, the accuracy, sensitivity, and specificity were substantially increased when 5% of outlying observations were incorporated into the real (GMP-PPAR) dataset using the THCM^14,48^ method (Table 1). Therefore, the proposed rHCoClust outperformed HCoClust in the presence of outliers in both real and simulated data. The proposed rHCoClust can also efficiently cluster and co-cluster the observations when missing observations in the dataset are replaced by outlying observations.

We also investigated the applicability of the rHCoClust approach for analyzing real-world toxicogenomic data using the three datasets (GMP-PPAR, GMP, and PPAR-SP) that were previously discussed. According to Table S1 and Fig. S10, the GMP-PPAR dataset’s important upregulatory co-clusters were (GC1, DCC1), (GC2, DCC1), and (GC3, DCC2). The PPAR-SP dataset’s major upregulatory co-cluster (GC1, DCC1), as shown in Figs. 5B, and 6D was nearly 100% comparable to the GMP-PPAR’s co-clusters (GC1, DCC1) and (GC2, DCC1) (Tables S2 and S4). Similarly, Tables S2 and S3 show that the significant co-cluster (GC3, DCC2) of the GMP-PPAR dataset was nearly 100% comparable to the co-clusters (GC1, DCC1) of the GMP dataset. By comparing these findings, it is possible to conclude that, in the case of real-world data, the rHCoClust method can also extract the actual co-clusters or patterns in the DCs and genes concurrently, just like it can in the case of simulated data. In the case of GMP and PPAR-SP datasets, rHCoClust identified two clusters of top-ranked DEGs (*GSTA5, MGST2, GCLC, GCLM, G6PD*) and (*EHHADH, CYP4A1, ANGPTL4, CPT1A*) that were highly regulated by two clusters of top-ranked DCs (acetaminophen_High _24.hr, nitrofurazone_High_24.hr, methapyrilene_High_24.hr) and (WY.14643_High_24.hr, clofibrate_High_24.hr, gemfibrozil_High_24.hr, benzbromarone_High_24.hr, aspirin_High_24.hr) respectively. The literature and functional annotation were used to validate the biomarker co-clusters generated by the rHCoClust algorithm. The toxicogenomic biomarker genes discovered by the suggested method were statistically highly significant in the corresponding pathways and DCs exhibiting toxicity evidence^7,57,62^. Significant biomarker genes in the co-cluster (GC1, DCC1) of the GMP dataset include *GSTA5*, which detoxifies chemicals in the liver^78^. Abnormal expression of *GST(A1-A5)* has also been associated with a higher risk of clear cell renal cell carcinoma, ovarian cancer, and colorectal cancer^79^. Trimeric integral membrane protein *MGST2* is a member of the membrane-associated proteins in the glutathione and eicosanoid metabolism family^80^. Glutamate cysteine ligase (*GCL*), a rate-limiting enzyme found in every mammalian tissue, is made up of a modifier (*GCLM*) and a catalytic (*GCLC*) subunit. It is essential for the detoxification of xenobiotics and serves as a defense against oxidative stress^81^. Glucose 6 phosphate dehydrogenase (*G6PD*) deficiency can lead to extensive intravascular hemolysis during acute viral hepatitis, which can induce acute kidney injury^82^. On the other hand, the important genes in the PPAR-SP dataset’s biomarker co-cluster (GC1, DCC1) are essential to the growth of PPARs. For example, *EHHADH* is critical for the metabolism of medium-chain dicarboxylic acids and suggests that hepatic cholesterol production is regulated by peroxisomal dicarboxylic acid β-oxidation^83^. The liver and kidney have high expression levels of the fatty acid and prostaglandin hydroxylase enzymes, often known as cytochrome P450 4A (*CYP4A*). The expression of the *CYP4A* genes in the liver and kidneys can be strongly stimulated by a wide range of substances known as peroxisome proliferators^84,85^. PPARs control the expression of *ANGPTL4*, which is extensively expressed in the liver and adipose tissue and is important in lipid metabolism^86^. Since *CPT1A* is the rate-limiting enzyme in fatty acid β-oxidation, deficiencies or aberrant regulation of this enzyme can lead to a variety of illnesses, including malignancies and metabolic disorders, making it a promising drug target for treatment^87^. Thus, the suggested rHCoClust algorithm might be utilized to analyze the biomarkers and chemical regulators since the DEGs and their DC regulators in the biomarker co-clusters comprise of biologically important biomarker genes and their regulatory DCs with particular mechanisms of action. The rHCoClust can be useful in other scientific domains including bioinformatics.

## Conclusions

Toxicogenomics is undoubtedly a useful tool for assessing the safety of medications and other substances. In this regard, toxogenomic biomarkers are utilized in this context for the assessment and prediction of toxicities, which are identified by computational methods on omics data from animal models. Finding toxicogenomic biomarkers in animal models has certain challenges and limitations, as these biomarkers occasionally may not accurately predict toxicity in humans. However, when it comes to accomplishing the goals of toxicogenomic research, conventional computational methods such as the t-test, SAM, LIMMA, ANOVA, bi-clustering, and machine learning have shortcomings. To address these limitations in this study, we proposed a robust hierarchical co-clustering approach to explore toxicogenomic biomarkers and associated chemical regulators. The proposed rHCoClust outperformed traditional HCoClust, since rHCoClust produced lower ER compared to HCoClust in the presence of outliers in the simulated dataset otherwise they perform equally. For real data, rHCoClust produced somewhat higher values of accuracy, sensitivity, and specificity for three ML methods RF, SVM, and GBM. However, if we modify 5% of data with THCM-based outliers^14,48^, rHCoClust shows significantly better results compared to HCoClust. Furthermore, since bi-clustering methods only function when the number of GCs and DCCs is equal and lack a criterion for detecting upregulatory and downregulatory co-clusters, rHCoClust outperformed bi-clustering approaches in detecting co-clusters when the number of GCs and DCCs is equal or not. Therefore, the proposed approach is not only useful for co-clustering and identification of biomarker co-clusters but also can be utilized for usual hierarchical clustering from robust viewpoints in any areas of bioinformatics and data sciences. To implement the proposed method easily by the researchers, we developed an R-package named ‘rhcoclust’, which can be downloaded from CRAN mirrors (https://cran.r-project.org/web/packages/rhcoclust/index.html).

## Supplementary Information

Supplementary Information.

## Data Availability

1. To implement the proposed method, we developed an R-package: Project name/software name: “rhcoclust” Project/software home page: https://cran.r-project.org/web/packages/rhcoclust/index.html Operating system: Platform independent Programing language: “R” 2. The preprint or trial version of the manuscript is available at BioRxiv, The preprint server of biology link: Link: https://www.biorxiv.org/content/10.1101/2020.05.13.094946v1. 3. Real data availability: Real data is available at: “https://toxygates.nibiohn.go.jp/toxygates/#columns” Real data is also accessible from the developed “R” package/software “rhcoclust”: https://cran.r-project.org/web/packages/rhcoclust/index.html.
