# Supplementary material for: Robust hierarchical co-clustering for exploring toxicogenomic biomarkers and their chemical regulators
Source: Sci Rep. 2025 May 14;15:16676. doi: 10.1038/s41598-025-99568-7 (PMC12078728; doi:10.1038/s41598-025-99568-7)
Supplement: Supplementary file 1 — Supplementary Information. [file 41598_2025_99568_MOESM1_ESM.rtf]

Supplementary Material 
Robust Hierarchical Co-Clustering for Exploring Toxicogenomic Biomarkers and Their Chemical Regulators
Mohammad Nazmol Hasan^1^, Md. Bahadur Badsha^2^ and Md. Nurul Haque Mollah^3*^  
^1^Department of Statistics, Gazipur Agricultural University, Gazipur-1706, Bangladesh. 
^2^Center for Applied Bioinformatics, St Jude Children's Research Hospital, Memphis, TN, United States.
†Present address: Sera Prognostics, Inc., Salt Lake City, UT, United States.
 Email: badshabio15@gmail.com
^3^Bioinformatics Lab., Department of Statistics, University of Rajshahi, Rajshahi-6205, Bangladesh.
 
*Correspondence: Md. Nurul Haque Mollah (mollah.stat.bio@ru.ac.bd)  


Contents:

Supplementary Sections (pages 2-6)
Supplementary Figures (pages 7-16)
Supplementary Tables (pages 17-28)


Supplementary Sections
S1. Hierarchical Clustering Algorithm 
Hierarchical clustering (HC) algorithms perform clustering with the row or column entities of a data matrix based on different distance and linkage/clustering methods. The distance method determines how the distance between two vector observations is calculated. Different linkage approaches are used to merge the row or column entities into clusters of data matrix based on the distance matrix. It builds clusters by repeatedly joining and merging the objects separated by the shortest distance. Following merging of the closest two points the distance matrix is updated and the process repeated until all objects are joined. The performance of the proposed robust hierarchical co-clustering (rHCoClust) algorithm and traditional HCoClust algorithm were compared based on three distance methods (euclidean, manhattan, minkowski) together with two linkage methods (ward.D, ward.D2) because in combination with these distance and linkage methods HCoClust outperform over other combinations of distance and linkage methods [1] . However, our proposed rHCoClust algorithm as well as the developed r-package "rhcoclust" are flexible in choosing the distance ("manhattan", "euclidean", "maximum", "canberra", "binary" or "minkowski") and agglomerative clustering or linkage ("ward.D", "ward.D2", "single", "complete", "average", "mcquitty", "median"  or "centroid") methods
S1.1. Distance Measures
The distance measure quantiﬁes the distance or dissimilarity among C-dimensional objects or items of a  data matrix. This method determines how the distance between two observations is calculated. In this study we considered  gene-DC toxicogenomic data matrix consisting of G =  genes and DC =  dose of chemicals (DCs)/treatment combinations. As mentioned earlier we transformed (logistic transformation) the gene-DC data matrix for robust results from the rHCoClust algorithm. Each of the inputs of this transformed data matrix is termed as  for the convenience of further use. This represents the transformed average FCGEs (average over animal sample) value for the gene under the treatment combination. In our rHCoClust algorithm, we consider Euclidean, Minkowski, Manhattan, Canberra, and Maximum distance methods [2,3]. 


S1.2. Different Linkages for Agglomerative Clustering  
The agglomerative clustering method is a hierarchical clustering technique that involves the gradual merging of smaller clusters to establish a hierarchy of clusters. The resulting clustering structure is considerably influenced by the choice of linkage criteria, which significantly influences the computation of the distance between clusters. The linkage methods single linkage, complete linkage, average linkage, centroid, median, or Ward's algorithm (“ward.D", "ward.D2) could be used in rHCoClust [2–4].  

S2. Simulated Data Contamination by Outliers 
An outlier is an observation in the dataset that arises for some unusual circumstances that deviate from the actual value of the observations. It is a common problem for gene expression/fold change gene expression data analysis. To compare the performance of the rHCoClust approaches with conventional hierarchical co-clustering (HCoClust) approaches in the absence and presence of outlying observations in the dataset, we contaminate the simulated FCGE dataset inputting outliers. The outlying observations in the simulated dataset are defined as five times larger FCGE values than the maximum value in the original dataset. These outlying observations may arise in the dataset casewise following the Tukey-Huber contamination model (THCM) [5,6] or independent cellwise following the independent contamination model (ICM) [5,7]. In our gene-DC FCGE toxicogenomic data matrix, each gene in the row is a case and each input in the data matrix is a cell. Therefore, for investigating the robustness of the proposed RHCoClust approaches over the conventional HC approaches we have contaminated the simulated dataset by outliers casewise and independent cellwise. The description of the THCM and ICM are given below. 

S2.1. Tukey-Huber Contamination Model (THCM)      
To examine the robustness of the proposed method we have contaminated the simulated gene-DCs FCGE dataset casewise/genewise using the following THCM: 
;  
Where  is the small proportion of cases (genes) to be contaminated,  is the distribution of the FCGEs of the outlier contaminated genes,  is the nominal distribution by which we have generated the simulated data. In this study, to measure the robustness of the proposed algorithm we have considered wide range of values of  (5%-40%). 
S2.2. Independent Contamination Model (ICM)
We also have contaminated the simulated gene-DCs FCGE dataset following ICM: 
 
Where , ,  is a  identity matrix, and  are independent  it indicates that  is the probability of each component in  to be contaminated. Additionally, the probability  indicates that at least one component in  to be contaminated. Where . In this study we consider the value of  are 0.000, 0.165, 0.304, 0.420, 0.517 and 0.598.
S3. Statistical Control Chart (SCC)
The control chart for individual measurement (CCIM) is a special type of statistical control chart (SCC) preferable for individual measurement or when the sample size is one for observing the quality characteristics of a product in a production process. The CCIM contains a central line (CL) which is the average value of the observations of quality characteristics, an upper control limit (UCL) and a lower control limit (LCL).  CCIM or a control chart has a close connection with the hypothesis testing for drawing inferences. If an observation plots outside the control limits (UCL or LCL) it indicates that the process running with assignable causes of variation which is equivalent to rejecting the null hypothesis of statistical control and immediate action is necessary to eliminate that causes for quality output. Consequently, an observation plotting within the control limits is equivalent to accept the hypothesis of statistical control and no action is necessary for quality output [8]  (Montgomery, 2016). In fact, there is an analogy between the process monitoring consisting of individual measurement and assessing the DCs/drugs toxicity based on toxicogenomic data [9] (Hasan et al., 2019a). We consider each of the co-cluster mean of FCGE data as individual measurement to explore the biomarker co-clusters. Accordingly, any co-cluster mean falling outside the limit UCL or LCL we consider that co-cluster as the biomarker co-cluster and GC and DCC that make this co-cluster are the clusters of toxicogenomic biomarkers and their regulatory DCs. On the other side, the co-clusters for which mean belong within the control limit (UCL and LCL) then the GCs and DCCs which make these co-clusters are the clusters of non-biomarker genes and safe DCs. In this regard we can calculate the CL, UCL and LCL in the following way.     

Suppose there are  clusters in the gens or row entity and  clusters in the DCs or column entity in the data matrix. Thus, according to proposed robust hierarchical co-clustering (rHCoClust) algorithm the GCs and DCCs together with make  co-clusters. If we consider the  is the mean of the  co-cluster, then for our problem the observations/measurements are . Using these observations, the CL, UCL and LCL can be calculated as:  


 

Where  is the moving range of two observations and if the moving range is calculated based on two observations, then  [8]. In this study, we have applied the SCC approach to the rHCoClust processed data to identify the biomarker co-clusters with toxicogenomic biomarker genes and their regulatory DCs. In this regard, this study used simulated and real (GMP and PPAR-SP) datasets from the online platform "Toxygates" [10] (https://toxygates.nibiohn.go.jp/toxygates/#columns) to show the applicability of the SCC in toxicogenomic data analysis. The CCIM-like approach “logistic moving range chart” is also applied for the identification of significant associations between toxicogenomic biomarkers and their regulatory DCs [9].  

References 
1. Hasan MN, Malek MB, Begum AA, Rahman M, Mollah MNH. Assessment of drugs toxicity and associated biomarker genes using hierarchical clustering. Med. 2019;55. 
2. Nielsen F. Introduction to HPC with MPI for Data Science. Springer. 2016; 
3. Johnshon RA, Wichern DW. Applied Multivariate Statistical Analysis 6th Edition.pdf. Technometrics. 2003;47. 
4. Murtagh F, Legendre P. Ward's Hierarchical Agglomerative Clustering Method: Which Algorithms Implement Ward's Criterion? J Classif. 2014;31. 
5. Hasan MN, Rana MM, Begum AA, Rahman M, Mollah MNH. Robust Co-clustering to Discover Toxicogenomic Biomarkers and Their Regulatory Doses of Chemical Compounds Using Logistic Probabilistic Hidden Variable Model. Front Genet. 2018;9. 
6. Agostinelli C, Leung A, Yohai VJ, Zamar RH. Robust estimation of multivariate location and scatter in the presence of cellwise and casewise contamination. Test. 2015;24. 
7. Alqallaf F, Van Aelst S, Yohai VJ, Zamar RH. Propagation of outliers in multivariate data. Ann Stat. 2009;37. 
8. Montgomery DC. Statistical quality control: a modern introduction. Stat. Qual. Control. 2013. 
9. Hasan MN, Begum AA, Rahman M, Haque Mollah MN. Robust identification of significant interactions between toxicogenomic biomarkers and their regulatory chemical compounds using logistic moving range chart. Comput Biol Chem. 2019;78. 
10. Nyström-Persson J, Igarashi Y, Ito M, Morita M, Nakatsu N, Yamada H, et al. Toxygates: Interactive toxicity analysis on a hybrid microarray and linked data platform. Bioinformatics. 2013;29. 


Supplementary Figures: 

Figure S1. Simulated data structure along with GCs, DCCs, and their co-clusters. (A) Simulated data structure, (B) Mixed data structure of (A), (C) Dendrogram of GCs derived from mixed data (B), and (D) Dendrogram of DCCs derived from mixed data (B). 

Figure S2. Hierarchical clustering using the distance and HC method combination manhattan: ward.D for the real datasets. (A) Gene clustering of GMP data. (B) Gene clustering of PPAR-SP data. (C) DCs clustering GMP data. (D) DCs clustering PPAR-SP data. 


Figure S3. Performance investigation of the proposed rHCoClustlust against the conventional HCoClust by plotting the average percentage of miss clustering error rates (ERs) against the different levels of outlying observations for 100 simulated datasets. In the graph MAN=manhattan, MIN=minkowski, EUC=Euclidean, WD=Words'method1, WD2= Words'method2 and =probability of at least one component of the dataset contaminated by outliers. (A) ERs of genes clustering in the presence of different levels of THCM-based outliers. (B) ERs of genes clustering in the presence of different levels of ICM-based outliers.  (C) ERs of DCs clustering in varying levels of THCM-based outliers. (D) ERs of DCs clustering in varying levels of ICM-based outliers.


Figure S4. Schematic representation of the co-clusters of the classical HCoClust and proposed rHCoClust algorithms based on the simulated data structures in the absence and presence of 10% outliers. (A) and (A*) represent the original data structures in the absence and presence of 10% outliers, respectively. (B) and (B*) represent the mixture of datasets obtained by the randomly allocated (gene, DC) original data structure in the absence and presence of 10% outliers, respectively.  (C) and (C*) represent the reconstructed data structures from the mixture datasets in the absence and presence of 10% outliers respectively, by the classical HCoClust. (D) and (D*) represent the reconstructed data structures from the mixture datasets in the absence and presence of 10% outliers, respectively, by the proposed rHCoClustlust. 

Figure S5. The plot of accuracy was used to compare the clustering stability of the first real dataset using the suggested rHCoClust and HCoClust approaches. In the figure, 50 iterated accuracy results produced by the rHCoClust approach are represented by the red-colored boxplot (or letter T' after ML methods in the horizontal axis), and 50 iterated accuracy results produced by the HCoClust approaches are represented by the green-colored boxplot (or letter 'R' after ML methods in the horizontal axis). (A) and (B), respectively, show the DCC and GC's clustering stability or accuracy.


Figure S6. Co-cluster graph of the simulated dataset generated using rHCoClust. In the graph, GCs are plotted along the X-axis, and DCCs are plotted along the Y-axis. Each row cluster in the X-axis makes a co-cluster with the column cluster in the Y-axis.   


Figure S7. Statistical control chart (SCC) to identify biomarker co-cluster for the simulated dataset. The co-clusters (a combination of GC and DCC makes a co-cluster) mean which plots beyond the UCL or LCL are considered as the biomarker co-clusters. Accordingly, in the figure number of GC and DCC combinations (1,1) and (2, 2) are up-regulatory, and (1, 2) and (4, 1) are down-regulatory biomarker co-clusters. 

Figure S8. (A) For GMP dataset, R1-C1 red edge indicates significant up-regulatory co-cluster network between GC1 and DCC1, the other edges indicate un-regulatory co-clusters network between GCs and DCCs, (B) For PPAR-SP dataset, R1-C1 red edge indicates significant up-regulatory co-cluster network between GC1 and DCC1, the other edges indicate un-regulatory co-clusters network between GCs and DCCs,  (C) For simulated dataset, R1-C1 and R2-C2 red edges indicate significant up-regulatory co-cluster networks between GC1 and DCC1 & GC2 and DCC2, respectively, R1-C2 and R4-C1 blue edges indicate significant down-regulatory co-cluster networks between GC1 and DCC2 & GC4 and DCC1, respectively. The other edges indicate un-regulatory co-cluster networks between GCs and DCCs  
 Figure S9. (A) represents Co-cluster networks between members of row clusters (GCs) and column clusters (DCCs) of simulated data with black edges indicate insignificant (un-regulatory) co-cluster networks, co-cluster networks between members of GCs and DCCs with red edges indicate significant up-regulatory co-cluster networks and co-cluster networks between members of GCs and DCCs with blue edges indicate significant down-regulatory co-cluster networks.   (B) Up-regulatory (red edges) and down-regulatory (blue edges) co-cluster networks were separated from (A).

Figure S10. Co-clustered data structure recovered by the proposed rHCoClust method for the GMP-PPAR dataset. 

Figure S11. Statistical control chart (SCC) for biomarker co-cluster identification for GMP-PPAR dataset. The co-clusters (a combination of GC and DCC makes a co-cluster) mean which plots beyond the UCL or LCL are considered as the biomarker co-clusters. Accordingly, in the figure number of GC and DCC combinations (1,1), (2, 1), and (3, 2) are the up-regulatory, and (5, 1) are the down-regulatory biomarker co-clusters. 

Figure S12. (A) represents co-cluster networks between members of row clusters (GCs) and column clusters (DCCs) of the GMP-PPAR dataset with black edges indicate insignificant (un-regulatory) co-cluster networks, co-cluster networks between members of GCs and DCCs with red edges indicate significant up-regulatory co-cluster networks and co-cluster networks between members of GCs and DCCs with blue edges indicate significant down-regulatory co-cluster networks. (B) represents significant up-regulatory (red edges) and down-regulatory (blue edges) co-cluster networks that were separated from (A).

Figure S13. Co-cluster view of the simulated data generated from rHCoClust, BCBimax, BCCC, BCPlaid, BCQuest, BCQuestmet, BCQuesttord, and BCSpectral algorithms.   
Supplementary tables: 
Table S1: Distance measures/methods that were considered for the rHCoClust algorithm. 
	
Distance Method 	Mathematical Formulation	
Euclidean 		
Minkowski		
Manhattan		
Canbera		
Maximum		
   


Table S2. The gene and DC cluster numbers along with their ranked co-cluster mean for the GMP-PPAR real dataset produced by the rHCoClust algorithm. 

GC#, DCC# 	aLFCGEs	Co-cluster Significance 	
1,1  	0.8318437	Sig Up-Reg	
2,1  	0.6377166	Sig Up-Reg	
3,2  	0.6269181	Sig Up-Reg	
1,3  	0.6077310	Insignificance 	
3,1  	0.5161072	Insignificance	
2,3  	0.5065987	Insignificance	
4,1  	0.5062043	Insignificance	
3,3  	0.5037181	Insignificance	
4,3  	0.5015726	Insignificance	
1,2  	0.4957425	Insignificance	
4,2   	0.4897193	Insignificance	
5,2   	0.4819434	Insignificance	
5,3  	0.4796307	Insignificance	
2,2  	0.4756042	Insignificance	
5,1  	0.4616315	Sig Down-Reg	
Note: Significant up-regulatory co-clusters (Sig Up-Reg) and Significant down-regulatory co-clusters (Sig Down-Reg).  

Table S3. The gene and DC clusters (GCs and DCCs) of the GMP-PPAR dataset were retrieved by the proposed rHCoClust algorithm.  
GC #	Genes 	DCC #	DCs	
GC1
(Significantly up-regulated by DCC1)	ACAA1A, CPT1B, CYP4A1,   CYP4A2, CPT2, ANGPTL4,    CYP4A3, PLIN2, EHHADH, PLIN5, LOC100910385
	DCC1 (Significantly up-regulate GC1 and GC2 and down down-regulate GC5)	aspirin.High.24hr.Rat.Liver, aspirin.High.24hr.Rat.Liver,  aspirin.High.24hr.Rat.Liver, aspirin.Middle.24hr.Rat.Liver, aspirin.Middle.24hr.Rat.Liver, aspirin.Middle.24hr.Rat.Liver, aspirin.Low.24hr.Rat.Liver, aspirin.Low.24 hr.Rat.Liver, aspirin.Low.24hr.Rat.Liver, WY-14643.High.24 hr.Rat.Liver, 
WY-14643.High.24 hr.Rat.Liver, WY-14643.High.24 hr.Rat.Liver,       
WY-14643.Middle.24 hr.Rat.Liver, WY-14643.Middle.24 hr.Rat.Liver,     
WY-14643.Middle.24 hr.Rat.Liver, WY-14643.Low.24 hr.Rat.Liver, WY-14643.Low.24 hr.Rat.Liver, WY-14643.Low.24 hr.Rat.Liver, WY-14643.High.9 hr.Rat.Liver, WY-14643.High.9 hr.Rat.Liver, WY-14643.High.9 hr.Rat.Liver, WY-14643.Middle.9 hr.Rat.Liver, WY-14643.Middle.9 hr.Rat.Liver, WY-14643.Middle.9 hr.Rat.Liver,      
WY-14643.Low.9 hr.Rat.Liver, WY-14643.Low.9 hr.Rat.Liver, WY-14643.Low.9 hr.Rat.Liver, clofibrate.High.9 hr.Rat.Liver, clofibrate.High.9 hr.Rat.Liver,  clofibrate.High.9 hr.Rat.Liver,  clofibrate.Middle.9 hr.Rat.Liver, clofibrate.Middle.9 hr.Rat.Liver, benzbromarone.High.9 hr.Rat.Liver, benzbromarone.High.9hr.Rat.Liver, benzbromarone.High.9 hr.Rat.Liver, benzbromarone.Middle.9 hr.Rat.Liver, benzbromarone.Middle.9 hr.Rat.Liver, benzbromarone.Middle.9 hr.Rat.Liver, benzbromarone.Low.9 hr.Rat.Liver,  benzbromarone.High.24 hr.Rat.Liver, benzbromarone.High.24 hr.Rat.Liver, benzbromarone.High.24 hr.Rat.Liver, WY-14643.High.6 hr.Rat.Liver, WY-14643.High.6 hr.Rat.Liver, WY-14643.High.6 hr.Rat.Liver, WY-14643.Middle.6 hr.Rat.Liver, WY-14643.Middle.6 hr.Rat.Liver, WY-14643.Middle.6 hr.Rat.Liver,  WY-14643.Low.6 hr.Rat.Liver, WY-14643.Low.6 hr.Rat.Liver, WY-14643.Low.6 hr.Rat.Liver, benzbromarone.High.6 hr.Rat.Liver, benzbromarone.High.6 hr.Rat.Liver, benzbromarone.High.6 hr.Rat.Liver, benzbromarone.Middle.6 hr.Rat.Liver, benzbromarone.Middle.6 hr.Rat.Liver, benzbromarone.Middle.6 hr.Rat.Liver, gemfibrozil.High.24 hr.Rat.Liver,  gemfibrozil.High.24 hr.Rat.Liver,  gemfibrozil.High.24 hr.Rat.Liver, gemfibrozil.Middle.24 hr.Rat.Liver, gemfibrozil.Middle.24 hr.Rat.Liver, 
gemfibrozil.Middle.24 hr.Rat.Liver, gemfibrozil.Low.24 hr.Rat.Liver, 
clofibrate.High.24 hr.Rat.Liver, clofibrate.High.24 hr.Rat.Liver, clofibrate.High.24 hr.Rat.Liver    	
GC2
(Significantly up-regulated by DCC1)	OPLAH, ANPEP,  AQP7, RXRG, FABP7,       CYP8B1,  LOC100365047,    LOC100365047,  CPT1A, CPT1A, ME1,            ME1, FABP3, FADS2, SORBS1, GK, GK,            ACSL3        
	DCC2
(Significantly up-regulate GC3)	methapyrilene.High.9 hr.Rat.Liver, methapyrilene.High.9 hr.Rat.Liver,  methapyrilene.High.9 hr.Rat.Liver, acetaminophen.High.24 hr.Rat.Liver,  acetaminophen.High.24 hr.Rat.Liver, acetaminophen.High.24 hr.Rat.Liver,  acetaminophen.Middle.24 hr.Rat.Liver, acetaminophen.Middle.24 hr.Rat.Liver, acetaminophen.Middle.24 hr.Rat.Liver, methapyrilene.High.24 hr.Rat.Liver, methapyrilene.High.24 hr.Rat.Liver, methapyrilene.High.24 hr.Rat.Liver, methapyrilene.High.6 hr.Rat.Liver, methapyrilene.High.6 hr.Rat.Liver, methapyrilene.High.6 hr.Rat.Liver, methapyrilene.Middle.6 hr.Rat.Liver, methapyrilene.Middle.6 hr.Rat.Liver, methapyrilene.Middle.6 hr.Rat.Liver, methapyrilene.Low.6 hr.Rat.Liver, methapyrilene.Low.6 hr.Rat.Liver, methapyrilene.Low.6 hr.Rat.Liver, nitrofurazone.High.24 hr.Rat.Liver, nitrofurazone.High.24 hr.Rat.Liver, nitrofurazone.High.24 hr.Rat.Liver, nitrofurazone.High.6 hr.Rat.Liver, nitrofurazone.High.6 hr.Rat.Liver, nitrofurazone.High.6 hr.Rat.Liver, nitrofurazone.Middle.6 hr.Rat.Liver, nitrofurazone.Middle.6 hr.Rat.Liver, nitrofurazone.Middle.6 hr.Rat.Liver, nitrofurazone.Low.6 hr.Rat.Liver, nitrofurazone.Low.6 hr.Rat.Liver, nitrofurazone.Low.6 hr.Rat.Liver, nitrofurazone.High.9 hr.Rat.Liver, nitrofurazone.High.9 hr.Rat.Liver, nitrofurazone.High.9 hr.Rat.Liver, nitrofurazone.Middle.9 hr.Rat.Liver, nitrofurazone.Middle.9 hr.Rat.Liver, nitrofurazone.Middle.9 hr.Rat.Liver, nitrofurazone.Low.9 hr.Rat.Liver, nitrofurazone.Low.9 hr.Rat.Liver, nitrofurazone.Low.9 hr.Rat.Liver, acetaminophen.High.9 hr.Rat.Liver, acetaminophen.High.9 hr.Rat.Liver, acetaminophen.High.9 hr.Rat.Liver, acetaminophen.Middle.9 hr.Rat.Liver, acetaminophen.Middle.9 hr.Rat.Liver, acetaminophen.Middle.9 hr.Rat.Liver, acetaminophen.Low.9 hr.Rat.Liver, acetaminophen.Low.9 hr.Rat.Liver, acetaminophen.Low.9 hr.Rat.Liver"    	
GC3
(Significantly up-regulated by DCC2)	LOC100912604/SRM, G6PD, GSTA2, GSTT1, GSR, GCLM, ODC1, GSS, GCLC, GSTA5,                           LOC100360180,                    RRM1, GCLC, MGST2,                           APITD1/CORT/KIF1B/LOC100360180, SMS, GSTM4, GSTP1, SMS, ACSL4                          	DCC3 (Insignificant)	methapyrilene.Middle.9 hr.Rat.Liver, methapyrilene.Middle.9 hr.Rat.Liver, methapyrilene.Middle.9 hr.Rat.Liver, methapyrilene.Low.9 hr.Rat.Liver, methapyrilene.Low.9 hr.Rat.Liver, methapyrilene.Low.9 hr.Rat.Liver, clofibrate.High.3 hr.Rat.Liver, clofibrate.High.3 hr.Rat.Liver, clofibrate.High.3 hr.Rat.Liver, clofibrate.Middle.3 hr.Rat.Liver, clofibrate.Middle.3 hr.Rat.Liver, clofibrate.Middle.3 hr.Rat.Liver, clofibrate.Low.3 hr.Rat.Liver , clofibrate.Low.3 hr.Rat.Liver, clofibrate.Low.3 hr.Rat.Liver, gemfibrozil.High.6 hr.Rat.Liver, gemfibrozil.High.6 hr.Rat.Liver, gemfibrozil.High.6 hr.Rat.Liver, gemfibrozil.Middle.6 hr.Rat.Liver, gemfibrozil.Middle.6 hr.Rat.Liver, gemfibrozil.Middle.6 hr.Rat.Liver, gemfibrozil.Low.6 hr.Rat.Liver, gemfibrozil.Low.6 hr.Rat.Liver, gemfibrozil.Low.6 hr.Rat.Liver, nitrofurazone.High.3 hr.Rat.Liver, nitrofurazone.High.3 hr.Rat.Liver, nitrofurazone.High.3 hr.Rat.Liver, nitrofurazone.Middle.3 hr.Rat.Liver, nitrofurazone.Middle.3 hr.Rat.Liver, nitrofurazone.Middle.3 hr.Rat.Liver, nitrofurazone.Low.3 hr.Rat.Liver, nitrofurazone.Low.3 hr.Rat.Liver, nitrofurazone.Low.3 hr.Rat.Liver, clofibrate.Middle.9 hr.Rat.Liver, clofibrate.Low.9 hr.Rat.Liver, clofibrate.Low.9 hr.Rat.Liver, clofibrate.Low.9 hr.Rat.Liver, acetaminophen.Low.24 hr.Rat.Liver, acetaminophen.Low.24 hr.Rat.Liver, acetaminophen.Low.24 hr.Rat.Liver, benzbromarone.High.3 hr.Rat.Liver, benzbromarone.High.3 hr.Rat.Liver, benzbromarone.High.3 hr.Rat.Liver, benzbromarone.Middle.3 hr.Rat.Liver, benzbromarone.Middle.3 hr.Rat.Liver, benzbromarone.Middle.3 hr.Rat.Liver, benzbromarone.Low.3 hr.Rat.Liver, benzbromarone.Low.3 hr.Rat.Liver, benzbromarone.Low.3 hr.Rat.Liver, benzbromarone.Low.9 hr.Rat.Liver, benzbromarone.Low.9 hr.Rat.Liver, methapyrilene.Middle.24 hr.Rat.Liver, methapyrilene.Middle.24 hr.Rat.Liver, methapyrilene.Middle.24 hr.Rat.Liver, methapyrilene.Low.24 hr.Rat.Liver, methapyrilene.Low.24 hr.Rat.Liver, methapyrilene.Low.24 hr.Rat.Liver, benzbromarone.Middle.24 hr.Rat.Liver, benzbromarone.Middle.24 hr.Rat.Liver, benzbromarone.Middle.24 hr.Rat.Liver, benzbromarone.Low.24 hr.Rat.Liver, benzbromarone.Low.24 hr.Rat.Liver, benzbromarone.Low.24 hr.Rat.Liver, nitrofurazone.Middle.24 hr.Rat.Liver, nitrofurazone.Middle.24 hr.Rat.Liver, nitrofurazone.Middle.24 hr.Rat.Liver, nitrofurazone.Low.24 hr.Rat.Liver, nitrofurazone.Low.24 hr.Rat.Liver, nitrofurazone.Low.24 hr.Rat.Liver, WY-14643.High.3 hr.Rat.Liver, WY-14643.High.3 hr.Rat.Liver, WY-14643.High.3 hr.Rat.Liver, WY-14643.Middle.3 hr.Rat.Liver, WY-14643.Middle.3 hr.Rat.Liver, WY-14643.Middle.3 hr.Rat.Liver, WY-14643.Low.3 hr.Rat.Liver, WY-14643.Low.3 hr.Rat.Liver, WY-14643.Low.3 hr.Rat.Liver, acetaminophen.High.6 hr.Rat.Liver, acetaminophen.High.6 hr.Rat.Liver, acetaminophen.High.6 hr.Rat.Liver, acetaminophen.Middle.6 hr.Rat.Liver, acetaminophen.Middle.6 hr.Rat.Liver, acetaminophen.Middle.6 hr.Rat.Liver, acetaminophen.Low.6 hr.Rat.Liver, acetaminophen.Low.6 hr.Rat.Liver, acetaminophen.Low.6 hr.Rat.Liver, aspirin.High.3 hr.Rat.Liver, aspirin.High.3 hr.Rat.Liver, aspirin.High.3 hr.Rat.Liver, aspirin.Middle.3 hr.Rat.Liver, aspirin.Middle.3 hr.Rat.Liver, aspirin.Middle.3 hr.Rat.Liver, aspirin.Low.3 hr.Rat.Liver, aspirin.Low.3 hr.Rat.Liver, aspirin.Low.3 hr.Rat.Liver, aspirin.High.6 hr.Rat.Liver, aspirin.High.6 hr.Rat.Liver, aspirin.High.6 hr.Rat.Liver, aspirin.Middle.6 hr.Rat.Liver, aspirin.Middle.6 hr.Rat.Liver, aspirin.Middle.6 hr.Rat.Liver, aspirin.Low.6 hr.Rat.Liver, aspirin.Low.6 hr.Rat.Liver, aspirin.Low.6 hr.Rat.Liver, aspirin.High.9 hr.Rat.Liver, aspirin.High.9 hr.Rat.Liver, aspirin.High.9 hr.Rat.Liver, aspirin.Middle.9 hr.Rat.Liver, aspirin.Middle.9 hr.Rat.Liver, aspirin.Middle.9 hr.Rat.Liver, aspirin.Low.9 hr.Rat.Liver, aspirin.Low.9 hr.Rat.Liver, aspirin.Low.9 hr.Rat.Liver, acetaminophen.High.3 hr.Rat.Liver, acetaminophen.High.3 hr.Rat.Liver, acetaminophen.High.3 hr.Rat.Liver,     acetaminophen.Middle.3 hr.Rat.Liver, acetaminophen.Middle.3 hr.Rat.Liver,   acetaminophen.Middle.3 hr.Rat.Liver, acetaminophen.Low.3 hr.Rat.Liver,     acetaminophen.Low.3 hr.Rat.Liver, acetaminophen.Low.3 hr.Rat.Liver,  clofibrate.High.6 hr.Rat.Liver, clofibrate.High.6 hr.Rat.Liver, clofibrate.High.6 hr.Rat.Liver, clofibrate.Middle.6 hr.Rat.Liver, clofibrate.Middle.6 hr.Rat.Liver, clofibrate.Middle.6 hr.Rat.Liver, clofibrate.Low.6 hr.Rat.Liver, clofibrate.Low.6 hr.Rat.Liver, clofibrate.Low.6 hr.Rat.Liver, benzbromarone.Low.6 hr.Rat.Liver, benzbromarone.Low.6 hr.Rat.Liver, benzbromarone.Low.6 hr.Rat.Liver, gemfibrozil.Low.24 hr.Rat.Liver, gemfibrozil.Low.24 hr.Rat.Liver, gemfibrozil.High.3 hr.Rat.Liver, gemfibrozil.High.3 hr.Rat.Liver, gemfibrozil.High.3 hr.Rat.Liver, gemfibrozil.Middle.3 hr.Rat.Liver, gemfibrozil.Middle.3 hr.Rat.Liver, gemfibrozil.Middle.3 hr.Rat.Liver, gemfibrozil.Low.3 hr.Rat.Liver, gemfibrozil.Low.3 hr.Rat.Liver, gemfibrozil.Low.3 hr.Rat.Liver, gemfibrozil.High.9 hr.Rat.Liver, gemfibrozil.High.9 hr.Rat.Liver, gemfibrozil.High.9 hr.Rat.Liver, gemfibrozil.Middle.9 hr.Rat.Liver, gemfibrozil.Middle.9 hr.Rat.Liver, gemfibrozil.Middle.9 hr.Rat.Liver, gemfibrozil.Low.9 hr.Rat.Liver, gemfibrozil.Low.9 hr.Rat.Liver, gemfibrozil.Low.9 hr.Rat.Liver, clofibrate.Middle.24 hr.Rat.Liver, clofibrate.Middle.24 hr.Rat.Liver, clofibrate.Middle.24 hr.Rat.Liver, clofibrate.Low.24 hr.Rat.Liver, clofibrate.Low.24 hr.Rat.Liver, clofibrate.Low.24 hr.Rat.Liver, methapyrilene.High.3 hr.Rat.Liver, methapyrilene.High.3 hr.Rat.Liver, methapyrilene.High.3 hr.Rat.Liver, methapyrilene.Middle.3 hr.Rat.Liver, methapyrilene.Middle.3 hr.Rat.Liver, methapyrilene.Middle.3 hr.Rat.Liver, methapyrilene.Low.3 hr.Rat.Liver, methapyrilene.Low.3 hr.Rat.Liver, methapyrilene.Low.3 hr.Rat.Liver    	
GC4
(Insignificant)	GPX1, MGST1, GSTA3, GSTT2, HPGDS, GPX3, IDH1, GSTM2, GPX7, GPX8, GSTA4, LAP3, GPX4, GSTM1, GSTM7,  APITD1/CORT/KIF1B/LOC100360180.2, MGST3, IDH2, TXNDC12, GSTO1, APITD1/CORT/KIF1B/LOC100360180.3, GGCT, GSTK1, ACADM, LOC100912469, PCK1, ILK, DBI, LPL, APOA1, ACSL1, ACSL1.2, ACADL, PCK2, CYP27A1, SLC27A5, SLC27A2, SCP2, SCP2, APOA2, ACOX3, HMGCS2, PPARD, RXRA, APOC3, APOA5, FABP1, UBB, SLC27A1, ACSL5, NR1H3                           	---	---	
GC5 (Significantly down-regulated by DCC1)	GGT7, GGT1, GSTM3, GSTM5/LOC100912430, GPX2, RGD1562107, GSTO2/LOC100909560, NAT8, APITD1/CORT/KIF1B/LOC100360180.1, GGT5, GPX6, LOC100359539/RRM2, GPX4.1, LOC100359539/RRM2, IDH1, GGT5, GSTT2, GGCT,  FABP2, LOC100912469, CPT1C, PLIN1, LOC100909612, SCD, PPARA, FABP6, CYP7A1, ACSL1, PCK2, PLTP, PLIN4, SCD2, FABP4, PDPK1, RXRB, PPARG, FABP5, CYP4A8, CYP4A8, ADIPOQ, ACOX2, PPARD, ACSBG1, ACSL6, SLC27A4, OLR1, PLTP, SCD, ACSL5, RXRG                       	---	---	

Table S4. The gene and DC clusters (GCs and DCCs respectively) of the GMP dataset were retrieved by the proposed rHCoClust algorithm.  
GC #	Genes 	DCC #	DCs	
GC1
(Significantly up-regulated by DCC1)	LOC100912604.SRM, GSTA2, GSTA5, GCLC,           GSTM4, G6PD, GSTA5,  GCLC, ODC1, MGST2,     GSR, GSS, GCLM, GSTP1          	DCC1
(Significantly up-regulate GC1)	acetaminophen_High_9.hr, acetaminophen_High_24.hr, acetaminophen_Middle_24.hr, acetaminophen_Low_9.hr,    
acetaminophen_Middle_9.hr, methapyrilene_High_6.hr,  
methapyrilene_High_24.hr, methapyrilene_High_9.hr,    
nitrofurazone_High_24.hr, nitrofurazone_High_6.hr,    
nitrofurazone_Middle_6.hr, nitrofurazone_Low_9.hr, nitrofurazone_Low_6.hr, nitrofurazone_Middle_24.hr, nitrofurazone_High_9.hr, nitrofurazone_Middle_9.hr 	
GC2
(Insignificant)	GPX7, GSTM7, GPX8,             LOC100360180, GGT1, GSTM5.LOC100912430, IDH1, GSTM1, MGST3, GSTA3, GSTA, APITD1, CORT. KIF1B, LOC100360180, GPX1, NAT8, ANPEP, GSTT1, LAP3, GSTO1, SMS, TXNDEC12, MGST1, GSTM2,   OPLAH, APITD1, CORT, KIF1B, LOC100360180, GSTT2, IDH2, SMS, GSTK1, RRM1, HPGDS, GPX3, APITD1. CORT. KIF1B, LOC100360180, GPX4                         	DCC2
(Insignificant)	acetaminophen_Middle_6.hr, acetaminophen_Low_24.hr, acetaminophen_Low_6.hr, acetaminophen_Middle_3.hr, acetaminophen_High_6.hr, hexachlorobenzene_High_24.hr", hexachlorobenzene_Middle_3.hr, hexachlorobenzene_Low_9.hr, hexachlorobenzene_Low_24.hr, hexachlorobenzene_High_6.hr, hexachlorobenzene_Low_6.hr, hexachlorobenzene_Middle_24.hr, hexachlorobenzene_High_9.hr, hexachlorobenzene_Middle_6.hr, hexachlorobenzene_Middle_9.hr, methapyrilene_Middle_24.hr, methapyrilene_Low_9.hr, methapyrilene_Middle_3.hr,  methapyrilene_High_3.hr, methapyrilene_Low_3.hr, methapyrilene_Low_6.hr, methapyrilene_Middle_9.hr, methapyrilene_Low_24.hr, methapyrilene_Middle_6.hr, nitrofurazone_Low_3.hr, nitrofurazone_High_3.hr, nitrofurazone_Low_24.hr, nitrofurazone_Middle_3.hr, erythromycin_Middle_24.hr, erythromycin_High_24.hr, erythromycin_Low_24.hr, erythromycin_High_3.hr, erythromycin_Middle_3.hr, erythromycin_Low_3.hr, glibenclamide_High_3.hr, glibenclamide_Low_9.hr, glibenclamide_High_9.hr, glibenclamide_High_6.hr, glibenclamide_Middle_9.hr, glibenclamide_Low_3.hr, glibenclamide_Middle_6.hr, glibenclamide_Low_6.hr, glibenclamide_Middle_3.hr, isoniazid_Low_24.hr, isoniazid_Low_6.hr, isoniazid_Middle_6.hr, isoniazid_High_9.hr, isoniazid_High_6.hr, isoniazid_High_3.hr, perhexiline_Low_9.hr, perhexiline_Middle_3.hr, perhexiline_High_9.hr, perhexiline_Low_3.hr, perhexiline_High_3.hr, perhexiline_Middle_9.hr, gentamicin_Middle_24.hr, gentamicin_Middle_9.hr, gentamicin_Middle_3.hr, gentamicin_Low_24.hr, gentamicin_High_24.hr, gentamicin_High_9.hr, gentamicin_Low_3.hr, gentamicin_High_3.hr, gentamicin_Low_9.hr, penicillamine_High_24.hr, penicillamine_Low_6.hr, penicillamine_Middle_6.hr, penicillamine_High_6.hr     	
GC3
(Insignificant)	GGCT, GPX4, APITD1.CORT. KIF1B.LOC100360180, Gstt2, LOC100359539.Rrm2, Ggt7, Gstm3, LOC100359539.Rrm2, Gsto2.LOC100909560, Idh1, Ggt5, Ggt5, Gpx2, Gpx6, RGD1562107                    	DCC3
(Insignificant)	acetaminophen_Low_3.hr, acetaminophen_High_3.hr, hexachlorobenzene_Low_3.hr, hexachlorobenzene_High_3.hr, erythromycin_Middle_9.hr, erythromycin_Low_9.hr,  
erythromycin_Middle_6.hr, erythromycin_High_6.hr, erythromycin_High_9.hr, erythromycin_Low_6.hr, glibenclamide_Low_24.hr, glibenclamide_High_24.hr, glibenclamide_Middle_24.hr, isoniazid_Low_3.hr, isoniazid_Middle_24.hr, isoniazid_Middle_9.hr, isoniazid_Low_9.hr, isoniazid_High_24.hr, isoniazid_Middle_3.hr, perhexiline_High_6.hr, perhexiline_Low_24.hr, perhexiline_High_24.hr, perhexiline_Low_6.hr, perhexiline_Middle_6.hr, perhexiline_Middle_24.hr, gentamicin_High_6.hr, gentamicin_Low_6.hr, gentamicin_Middle_6.hr, penicillamine_Low_3.hr, penicillamine_High_3.hr, penicillamine_Low_24.hr, penicillamine_High_9.hr, penicillamine_Low_9.hr, penicillamine_Middle_3.hr, penicillamine_Middle_9.hr, penicillamine_Middle_24.hr	


Table S5. The GCs and DCCs members of PPAR signaling pathway data retrieved by the rHCoClust co-clustering algorithm.  
GC #	Genes	DCC #	DCs	
GC1
(Significantly up-regulated by DCC1)	CPT1A, CYP4A3, EHHADH, PLIN5, LOC100910385, CPT2, ACAA1A, CYP4A1, CPT1A, CYP4A2, ANGPTL4, CPT1B	DCC1
(Significantly up-regulate GC1)	aspirin_Low_24.hr, aspirin_High_24.hr, aspirin_Middle_24.hr, benzbromarone_Middle_6.hr, benzbromarone_High_9.hr, benzbromarone_Middle_9.hr, benzbromarone_High_24.hr, benzbromarone_High_6.hr, clofibrate_Middle_6.hr, clofibrate_High_24.hr, clofibrate_Middle_9.hr, clofibrate_High_6.hr, clofibrate_High_9.hr, gemfibrozil_High_24.hr, gemfibrozil_Middle_24.hr, gemfibrozil_Low_24.hr, WY.14643_High_6.hr, WY.14643_Middle_6.hr, WY.14643_Middle_24.hr, WY.14643_Low_3.hr, WY.14643_Low_24.hr, WY.14643_Middle_9.hr, WY.14643_Low_6.hr, WY.14643_High_9.hr, WY.14643_Middle_3.hr, WY.14643_High_3.hr, WY.14643_Low_9.hr, WY.14643_High_24.hr   	
GC2
(Insignificant)
	RXRA, CYP8B1, SORBS1, PLIN2, FABP2, SCP2, PCK2, HMGCS2, ACSL4, LOC100912469, PPARD, APOA5, FABP3, ACOX2, ME1, ACOX3, ACSL5, FADS2, SLC27A5, GK, ILK, SLC27A2, ACSL5, SCP2, RXRB, PPARG, DBI, LPL, FABP7, ACSL1, PDPK1, LOC100365047, 
APOC3, APOA2, GK, PCK1, ACSL1, SLC27A1, SCD2, PLTP, ACADL, PLTP, LOC100365047, FABP1, ACSL3, APOA1, RXRG, AQP7, UBB, RXRG, ACADM, CYP27A1, ME1, NR1H3         	DCC2
(Insignificant)	aspirin_High_3.hr, aspirin_Low_9.hr, aspirin_High_9.hr, aspirin_Low_3.hr, aspirin_Middle_9.hr, aspirin_Low_6.hr, aspirin_Middle_3.hr, aspirin_High_6.hr, aspirin_Middle_6.hr, benzbromarone_Low_3.hr, benzbromarone_High_3.hr, benzbromarone_Low_6.hr, benzbromarone_Middle_3.hr, benzbromarone_Low_9.hr, benzbromarone_Low_24.hr, benzbromarone_Middle_24.hr, clofibrate_Middle_3.hr, clofibrate_Middle_24.hr, clofibrate_High_3.hr, clofibrate_Low_6.hr, clofibrate_Low_3.hr, clofibrate_Low_9.hr, clofibrate_Low_24.hr, gemfibrozil_Low_9.hr, gemfibrozil_Middle_6.hr, gemfibrozil_High_9.hr, gemfibrozil_Low_6.hr, gemfibrozil_Middle_9.hr, gemfibrozil_High_3.hr, gemfibrozil_Middle_3.hr, gemfibrozil_High_6.hr     	
GC3
(Insignificant)
	OLR1, LOC100912469, ADIPOQ, CYP4A8, ACSL6, FABP5, SCD, FABP6, ACSBG1, PPARD, SCD, FABP4, ACSL1, PCK2, PPARA, PLIN1, PLIN4, CPT1C, CYP4A8, CYP7A1, LOC100909612, SLC27A4     	DCC3
(Insignificant)
	cisplatin_Middle_6.hr, cisplatin_Low_24.hr, cisplatin_High_3.hr, cisplatin_Middle_24.hr, cisplatin_Middle_3.hr, cisplatin_High_6.hr, 
cisplatin_Low_6.hr, cisplatin_Low_3.hr, cisplatin_High_9.hr, cisplatin_Low_9.hr, cisplatin_High_24.hr, cisplatin_Middle_9.hr, 
diltiazem_High_6.hr, diltiazem_Middle_3.hr, diltiazem_High_24.hr, diltiazem_High_3.hr, diltiazem_High_9.hr, diltiazem_Middle_6.hr, 
diltiazem_Low_9.hr, diltiazem_Low_6.hr, diltiazem_Low_24.hr, diltiazem_Middle_24.hr, diltiazem_Low_3.hr, diltiazem_Middle_9.hr, 
gemfibrozil_Low_3.hr, methapyrilene_Middle_24.hr, 
methapyrilene_Low_9.hr, methapyrilene_High_6.hr, 
methapyrilene_Middle_3.hr, methapyrilene_High_24.hr, 
methapyrilene_High_3.hr, methapyrilene_Low_3.hr, 
methapyrilene_Low_6.hr, methapyrilene_High_9.hr, 
methapyrilene_Middle_9.hr, methapyrilene_Low_24.hr, 
methapyrilene_Middle_6.hr, phenobarbital_Middle_3.hr, 
phenobarbital_Low_24.hr, phenobarbital_High_9.hr, 
phenobarbital_High_24.hr, phenobarbital_Middle_24.hr, 
phenobarbital_Middle_9.hr, phenobarbital_Low_9.hr, 
phenobarbital_Middle_6.hr, phenobarbital_Low_3.hr, 
phenobarbital_Low_6.hr, phenobarbital_High_6.hr, 
phenobarbital_High_3.hr, triazolam_High_24.hr, 
triazolam_High_9.hr, triazolam_Low_6.hr, 
triazolam_Middle_6.hr, triazolam_Middle_9.hr, 
triazolam_Low_24.hr, triazolam_Low_3.hr, triazolam_High_3.hr, triazolam_Middle_24.hr, triazolam_High_6.hr, triazolam_Middle_3.hr, 
triazolam_Low_9.hr        	

Table S6. Functional annotation of KEGG pathway on the biomarker genes (genes in biomarker co-cluster 1) explored by the rHCoClust algorithm for PPAR signaling pathway datasets.   
	Term	Count	%	p-value	Genes 	
PPAR Signaling Pathway	rno03320:PPAR signaling pathway	11	80.33	5.59E-20   	SORBS1, CPT1B, CPT2, 
EHHADH, ACSL3, ACAA1A, 
CYP4A2, CPT1A, CYP4A3, 
CPT1A, ANGPTL4, 
CYP4A1   	
	rno00071:Fatty acid degradation	9  	60.0    	1.59E-16  	CPT1B, CPT2, EHHADH, ACSL3, ACAA1A, CYP4A2, 
CPT1A, CYP4A3, CPT1A, 
CYP4A1	
	rno01212:Fatty acid metabolism  	6	40.0    	6.08E-9   	CPT1B, CPT2, EHHADH, 
ACSL3, ACAA1A, CPT1A, 
CPT1A	
	rno01100:Metabolic pathways	7	46.66	0.004	ME1, ME1, EHHADH, ACSL3, ACAA1A, CYP4A2, 
CYP4A3, 
CYP4A1	
	rno04920:Adipocytokine signaling pathway        	3	20.0    	0.005	CPT1B, ACSL3, CPT1A, 
CPT1A	
	rno00590:Arachidonic acid metabolism    	3	20.0    	0.005	CYP4A2, CYP4A3, 
CYP4A1	
	rno00830:Retinol metabolism     	3	20.0	0.006	CYP4A2, CYP4A3, 
CYP4A1	
	rno04146:Peroxisome     	3	20.0    	0.006	EHHADH, ACSL3, ACAA1A	
	rno04750:Inflammatory mediator regulation of TRP channels	3	20.0    	0.011	CYP4A2, CYP4A3, 
CYP4A1	
	rno04270:Vascular smooth muscle contraction     	3	20.0    	0.012	CYP4A2, CYP4A3, 
CYP4A1	
	rno00280:Valine, leucine and isoleucine degradation     	2	13.33	0.075	EHHADH, 
ACAA1A	
                           
Table S7. Gene and DC co-cluster/bi-cluster results of simulated data using the proposed rHCoClust, BCBimax, BCCC, BCPlaid, BCQuest, BCQuestmet, BCQuesttord, and BCSpectral algorithms.
Algorithm	Co-cluster (CoC) (#Gene cluster, #DC cluster)	# Gene, # DC	Co-cluster Significance 	Algorithm	Bi-cluster (BiC) (#Gene cluster, #DC cluster)	# Gene, # DC	
rHCoClust	CoC 1 (1,1)	10,10	Sig Up-Reg	BCBimax	BiC 1 (1,1)	20,10	
	CoC 2 (2,2)	10,10	Sig Up-Reg		BiC 2 (2,2)	20,10	
	CoC 3 (1,3)	10,16	Insignificance		BiC 3 (3,3)	10,20	
	CoC 4 (3,1)	20,10	Insignificance	BCCC	BiC 1 (1,1)	41,36	
	CoC 5 (4,3)	10,16	Insignificance		BiC 2 (2,2)	9,36	
	CoC 6 (2,3)	10,16	Insignificance	BCPlaid	BiC 1 (1,1)	10,10	
	CoC 7 (3,3)	20,16	Insignificance		BiC 2 (2,2)	10,10	
	CoC 8 (4,2)	10,10	Insignificance		BiC 3 (3,3)	16,15	
	CoC 9 (2,1)	10,10	Insignificance		BiC 4 (4,4)	14,10	
	CoC 10 (3,2)	20,10	Insignificance		BiC 5 (5,5)	17,9	
	CoC 11 (1,2)	10,10	Sig Down-Reg	BCQuest	No bi-cluster found 	
	CoC 12 (4,1)	20,10	Sig Down-Reg	BCQuestmet	BiC 1 (1,1)	24.12	
			BiC 2 (2,2)	9,13	
			BiC 3 (3,3)	10,12	
		BCQuestord	BiC 1 (1,1)	41,16	
		BCSpectral	BiC 1 (1,1)	10,10	
			BiC 2 (2,2)	10,10	
Note: Significant up-regulatory co-clusters (Sig Up-Reg) and Significant down-regulatory co-clusters (Sig Down-Reg).
